# Pyroptosis in health and disease: mechanisms, regulation and clinical perspective

**DOI:** 10.1038/s41392-024-01958-2

**Published:** 2024-09-20

**Authors:** Yifan Liu, Renjie Pan, Yuzhen Ouyang, Wangning Gu, Tengfei Xiao, Hongmin Yang, Ling Tang, Hui Wang, Bo Xiang, Pan Chen

**Affiliations:** 1 The Affiliated Cancer Hospital of Xiangya School of Medicine, Central South University/Hunan Cancer Hospital, Changsha, 410013 China; 2 Department of Oncology, Xiangya Hospital, Central South University, 87th Xiangya road, Changsha, 410008 Hunan province China; 3 Department of Neurology, Xiangya Hospital, Central South University, 87th Xiangya road, Changsha, 410008 Hunan province China

**Keywords:** Molecular medicine, Drug development, Translational research

## Abstract

Pyroptosis is a type of programmed cell death characterized by cell swelling and osmotic lysis, resulting in cytomembrane rupture and release of immunostimulatory components, which play a role in several pathological processes. Significant cellular responses to various stimuli involve the formation of inflammasomes, maturation of inflammatory caspases, and caspase-mediated cleavage of gasdermin. The function of pyroptosis in disease is complex but not a simple angelic or demonic role. While inflammatory diseases such as sepsis are associated with uncontrollable pyroptosis, the potent immune response induced by pyroptosis can be exploited as a therapeutic target for anti-tumor therapy. Thus, a comprehensive review of the role of pyroptosis in disease is crucial for further research and clinical translation from bench to bedside. In this review, we summarize the recent advancements in understanding the role of pyroptosis in disease, covering the related development history, molecular mechanisms including canonical, non-canonical, caspase 3/8, and granzyme-mediated pathways, and its regulatory function in health and multiple diseases. Moreover, this review also provides updates on promising therapeutic strategies by applying novel small molecule inhibitors and traditional medicines to regulate pyroptosis. The present dilemmas and future directions in the landscape of pyroptosis are also discussed from a clinical perspective, providing clues for scientists to develop novel drugs targeting pyroptosis.

## Introduction

Cell death is critical for homeostasis in the human body because it eliminates unwanted cells.^1^ Among the different cell death types, regulated cell death (RCD) is encoded by genetic information and is accompanied by normal cell senescence.^2^ The absence of RCD contributes to the initiation and progression of diseases, such as cancer, which is characterized by uncontrolled cell growth and immortality. Apart from immortalization, excessive cell death is also correlated with the development of disease. For example, some neurodegenerative diseases, such as Alzheimer’s disease, are associated with abnormal loss of neurons during the human aging process.^3^ Therefore, understanding the different RCD modes and methods of reprogramming cell death is vital for disease therapy innovations.

RCD results from complex cellular responses to different stimuli and includes multiple forms, such as apoptosis, necroptosis, pyroptosis, and ferroptosis.^4^ With the expansion of research on cell death building on previous scientific endeavors. Pyroptosis is regulated by inflammatory caspases, inflammasome formation, and gasdermin aggregation on the membrane, which is induced by pathogen-associated molecular patterns (PAMPs), damage-associated molecular patterns (DAMPs), and pathogen infection. Pyroptosis involves plasma membrane rupture, chromatin condensation, DNA fragmentation, intact nuclei, pore formation, cell swelling, and osmotic lysis. DAMPs such as interleukin (IL)-18, IL-1β, dsDNA, ATP, and high mobility group box 1 (HMGB1) are also released during pyroptosis.^5^

**Fig. 1:**
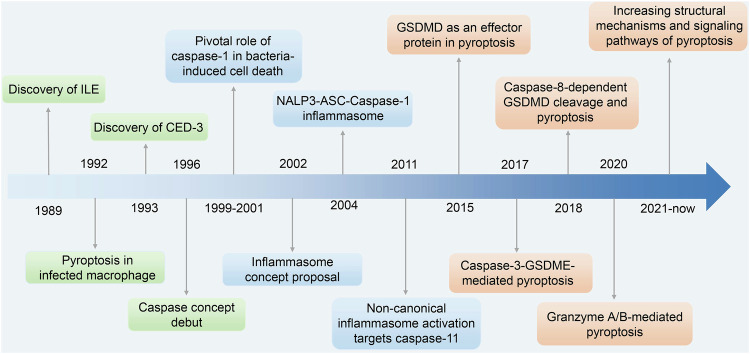
Milestones of research on the discovery and development of pyroptosis. From 1989 to 2001, pyroptosis was observed in cells, and related molecules such as IL-1β-converting enzyme (ILE) were identified. The inflammasome concept was formed and developed in the following decade. The discovery of GSDMD as an effector protein initiated the quick development of pyroptosis research with canonical, non-canonical, caspase-3/8 or granzyme-mediated pathways reported. Increasing pyroptosis studies related to specific diseases have also been performed in recent years

A holistic understanding of pyroptosis has been achieved after decades of exploration, as shown in Fig. 1. Notably, Kostura et al. and Black et al. first reported a novel enzyme called IL-1β-converting enzyme (ILE).^6,7^ In 1993, Yuan et al. reported that CED-3 shares similar functions with ILE.^8^ The ILE-CED3 enzyme family was named caspase in 1996 and is classified into two groups: inflammatory (caspase-1/4/5/11) and apoptotic (caspase-3/6/7, 2/8/9/10).^9^ The function and signaling pathways related to caspases were explored in subsequent years. However, pyroptosis and apoptosis were not distinguished from one another until later. For example, pyroptosis in infected macrophages was discovered in 1992 but was misclassified as apoptosis.^10^ Subsequent publications substantiated the pivotal and unique role of caspase-1 in bacterial-induced cell death.^11^ Several following studies proposed that caspase-1-mediated cell death differs from apoptosis because membrane integrity is destroyed by the inflammatory response.^11–13^ Later on, additional caspases, such as caspase-4/5/11, were found to be involved in what has now been coined “pyroptosis”.^14^ The term “pyroptosis” was first proposed in 2001 to describe this proinflammatory cell death type. Subsequently, Martinon et al. proposed the “inflammasome” concept in 2002, and further evidence identifying key components emerged in the following years, including NLRP1-CASP1 and NLRP3-ASC-CASP1.^15,16^ In addition to caspase-1, the function of caspase-11 in pyroptosis was reported in two studies by the Dixit group.^17,18^ In 2015, Feng et al. identified the effector proteins and showed that caspase-1/11/4/5 can cleave gasdermin D (GSDMD) to release its N-terminal domain.^19^ Dixit et al. and Han et al. proposed that GSDMD is an effector protein in pyroptosis.^20,21^ N-terminal GSDMD can aggregate on the membrane, forming holes that alter the intracellular osmotic pressure and lead to cell swelling until membrane fracture and cytokine release (e.g., IL-1β, IL-18).^22^ Shao et al. found that caspase-3 recognizes and hydrolyzes gasdermin E (GSDME) to induce pyroptosis.^23^ Other groups have reported a caspase-8-mediated pyroptosis pathway.^24,25^ These studies verified the hypothesis that the cell death pattern should not be classified by the caspase type but the substrate hydrolyzed by the caspase. Pyroptosis was classified as RCD in 2018 and is characterized by its reliance on the gasdermin protein family.^2^ In 2020, the mechanism through which granzyme A cleaves gasdermin B (GSDMB) and granzyme B cleaves GSDME to trigger pyroptosis were also discovered.^26,27^ Additional structural mechanisms and signaling pathways have been reported more recently.^28,29^

A close correlation between pyroptosis and disease has also been widely reported, and the gasdermin protein family’s functionality varies across cells.^30,31^ GSDMA is predominantly expressed within skin and epithelial tissues such as the hair follicle, epidermis, and gastric epithelium, which correlate to skin inflammation and alopecia.^32,33^ As for the GSDMB, it is widely expressed in the gastrointestinal epithelium, liver, and immune cells, which participates in the initiation and development of asthma and inflammatory bowel disease (IBD).^34,35^ The granzyme A released by CD8^+^ T and NK cells can activate GSDMB, leading to the pyroptosis.^27^ Furthermore, cancer cell pyroptosis was frequently activated by distinct molecular mechanisms relying on GSDMC and GSDME. The signal transduction after the binding of TNFα can activate the caspase-8/GSDMC pathway leading to tumor necrosis.^36^ In GSDME-expressing tumor cells, the granzyme B released from NK and CD8^+^ T lymphocytes and chimeric antigen receptor T (CAR-T) cells can cleave and activate caspase-3 and GSDME, triggering pyroptosis.^37^ Chemotherapy drugs also increase the expression of caspase-3 in GSDME cells, transform apoptosis into pyroptosis, and enhance anti-tumor immunity.^23^ However, higher GSDME expression level is correlated to unsatisfactory prognosis, indicating that GSDME may also possess the function of regulating cell proliferation rather than pyroptosis.^38^ Finally, GSDMD is the most widely researched protein of the gasdermin family, which can be activated by caspase-1/3/4/5/11, participating in multiple inflammatory diseases because of the immunogenic cellular content release during the pyroptosis.^39^ In macrophage and dendritic cells, a hyperactive caspase 1/GSDMD pathway correlates to excessive proinflammatory substance release (IL-1, IL-6, and tumor necrosis factor (TNF)-α) and an uncontrollable inflammatory status such as the cytokine release syndrome.^40^ From the perspective of treatment, small-molecule drugs targeting inflammasomes have increasingly emerged in the latest decade.^41^ Nanomaterials targeting the pyroptotic pathway have been increasingly developed and used in disease therapy.^42,43^ Therefore, as clinical scientists, in this review, we aimed to summarize the recent progress in the mechanism, regulation, and therapeutic perspectives of pyroptosis in disease. The current limitations and clinical dilemmas are also presented to inspire readers to propel further fundamental and clinical translational research.

## Molecular mechanisms of pyroptosis

Pyroptosis is a novel type of RCD with lytic and pro-inflammatory features. Pyroptotic and apoptotic cells both exhibit chromatin condensation and DNA fragmentation, but pyroptotic cells are distinguished by cell swelling, pore formation, osmotic lysis, and release of proinflammatory contents.^44–49^ Other unique forms of RCD, such as necroptosis and ferroptosis, have also recently emerged.^50–53^ Pyroptosis and necroptosis share the outcome of inflammatory stimuli release and immune responses, but the molecular pathways involved are different.^54–57^ Although iron can trigger both ferroptosis and iron-induced pyroptosis, ferroptosis is characterized by phospholipid peroxidation as a unique mode of cell death.^58–61^ A comparison of key aspects of apoptosis, necroptosis, ferroptosis, and pyroptosis, involving inducing factors, biochemical events, cell morphology, and cell release, is summarized in Table 1.

**Table 1:** Comparison between pyroptosis, apoptosis, necroptosis, and ferroptosis

Type	Pyroptosis	Apoptosis	Necroptosis	Ferroptosis
Inducing factors	ICD, Microbial infection, DAMPs, PAMPs,	TNF-α, FasL, TRAIL, Irradiation, Hypoxia, Heat shock	TNF-α, FasL, TRAIL, Pathogenic infection	Fe^2+^ overload initiation and ROS generation
Biochemical events	Assembly of inflammasome, Caspase-dependent GSDM cleavage, IL-18 and IL-1β release	HSPs, P53, Bcl-2 protein, cytochrome c release, formation of DISC, initiator/effector caspase cascade	RIPK1/RIPK3-mediated phosphorylation of MLKL, permeabilization of the lysosomal membrane, mitochondrial damage	iron accumulation, peroxidation of PUFAs, inhibition of Xc-system/GSH/GPX4
Cell morphology	Cells swelling, pore formation, rupture and bubbling of plasma membranes, Nucleus intact, Chromatin condensation, DNA fragmentation	Cell and nuclear volume reduced, Membrane intact, Formation of apoptotic bodies, Chromatin condensation, DNA fragmentation	Cells swelling, pore formation, rupture of plasma membranes, Cytoplasmic and organelle swelling, chromatin condensation	Cells swelling, pore formation, decreased mitochondria crista, Condensed mitochondrial membrane, Rupture of mitochondrial outer membrane
Cell release	Cellular content, DAMPs (IL-18, IL-10, IL-1β, HMGB1, ATP, dsDNA)	Usually no release, In certain cases: DAMPs (HMGB1, ATP, dsDNA, calreticulin)	Cellular content, DAMPs (IL-1α, IL-33, IL-6, HMGB1, ATP, HSPs)	DAMPs (KRAS-G12D, HMGB1, 8-OHG, and PGE2)
Inflammatory response	Yes	No	Yes	Yes
References	^22,47,57,75,109^	^48,49^	^54–57^	^53,58–61^

**Fig. 2:**
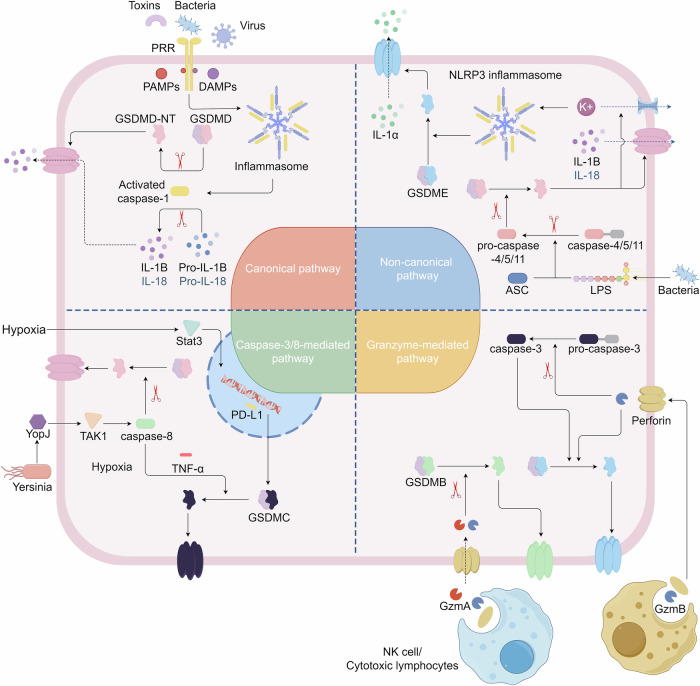
Signaling pathways of pyroptosis. In the canonical pathway of pyroptosis, PAMPs, and DAMPs are stimulated by intracellular signaling molecules. They combine with pro-caspase-1 and the adaptor protein ASC to form inflammasomes, leading to the activation of caspase-1. Cleaved caspase-1 then proceeds to cleave GSDMD and pro-IL-1β/IL-18. N-terminal GSDMD forms non-selective pores in the cell membrane, resulting in water influx, cell lysis, and ultimately cell death. Additionally, IL-1β and IL-18 are released through the pores formed by N-terminal GSDMD. In the non-canonical pathway, LPS activates caspase-4/5/11, triggering pyroptosis by cleaving GSDMD. The cleavage of GSDMD also leads to the efflux of K^+^, facilitating the assembly of the NLRP3 inflammasome and cleavage of pro-IL-1β and pro-IL-18. In the caspase-8-mediated pathway, the inhibition of TAK1 leads to the activation of caspase-8, which cleaves GSDMD, resulting in pyroptosis. Under hypoxic conditions, PD-L1 translocates to the nucleus and, in conjunction with phosphorylated Stat3, regulates the transcription of GSDMC, leading to the conversion of apoptosis to pyroptosis following TNFα-activated caspase-8. In the granzyme-mediated pathway, CAR-T cells rapidly activate caspase-3 in target cells by releasing GzmB. Subsequently, GSDME is activated, causing extensive pyroptosis. GzmA and GzmB from cytotoxic lymphocytes enter target cells through perforin and induce pyroptosis. GzmA hydrolyzes GSDMB, and GzmB directly activates GSDME. The figure was created by Figdraw

Characterization of pyroptosis in humans in 2015 revealed that the cleavage of GSDMD is primarily induced by inflammatory caspases, such as caspase-1, -4, -5, and -11.^19^ This cleavage results in the loss of interaction between the amino-terminal fragment and carboxy-terminal fragment in GSDMD. GSDMD belongs to the gasdermin superfamily, encompassing gasdermin A/B/C/D/E and DFNB59 (Pejvakin, PJVK) in humans and Gsdma1/2/3, Gsdmc1/2/3/4, Gsdmd, Dfna5, and Dfnb59 in mice. GSDME is also known as DFNA5.^20,22^ Each member comprises two conserved domains: the N-terminal pore-forming domain and the C-terminal repressor domain (PFD and RD), except DFNB59.^62–64^ Generally, the RD interacts with the PFD to maintain gasdermin oligomerization, suppressing its cytotoxic effect.^62,64^ However, when the PFD is separated from the RD, it assembles and forms perforations in the cell membrane in response to various internal and external stimuli. This process leads to the release of molecules associated with inflammation and pyroptosis.^22,65^ Therefore, gasdermin is regarded as an executor of pyroptosis. Several pathways that induce GSDMD cleavage have been identified, as shown in Fig. 2.

### Canonical pathway

Canonical pyroptosis is regulated by the integration of inflammasomes, which induce the cleavage of GSDMD and release of inflammatory factors such as IL-18 and IL-1β.^66–70^ Inflammasomes are activated and assembled to respond to microbial infections and endogenous danger signals, promoting host immune responses and cellular damage. During this process, released cell contents ultimately recruit innate immune cells to the infection site and modulate adaptive immune cells.^71–73^

The congregation of inflammasomes is initiated when cytosolic pattern recognition receptors (PRRs), known as detectors of inflammasomes, identify PAMPs and DAMPs.^30,74,75^ The activation of PRRs triggers downstream signaling cascades, leading to the presentation of type I interferons as well as the release of proinflammatory cytokines.^71,76–78^ Upon detection of bacterial or viral signals, PRRs assemble with pro-caspase-1 and adapter apoptosis-associated speck-like proteins containing a caspase activation and recruitment domain (CARD) (ASC) to form inflammasomes.^15,79–81^

Most inflammasomes consist of three segments: (i) leucine-rich repeat-containing proteins (NLRs), (ii) ASC, and (iii) pro-caspase-1.^40^ NLRs are composed of leucine-rich repeats (LRRs), a NACHT nucleotide-binding domain, and either a pyrin or CARD domain (PYD) at the N-terminus, which categorizes them into NLRP or NLRC subtypes, respectively.^82–84^ NLRC family members possess one or more N-terminal CARDs, as exemplified by NLRC4, whereas NLRP proteins, such as NLRP1 and NLRP3, harbor N-terminal PYDs.^78,85,86^

Extensive studies have been performed to characterize NLRC4, including ligand detection by NLR family apoptosis inhibitory protein (NAIP), which is the initial step in NLRC4 inflammasome activation.^87,88^ Additionally, non-NLR proteins, including AIM2 and pyrin, possess inflammasome-forming capacity. ASC, containing PYD and CARD, mediates inflammasome interactions and pro-caspase-1 recruitment.^85^ Following inflammasome assembly, caspase-1 undergoes autocatalytic cleavage, leading to the generation of active mature enzymes.^89^ Caspase-1 has a crucial impact on cleaving GSDMD, creating membrane pores, releasing IL-18 and IL-1β, and ultimately generating cell swelling and pyroptosis.^90,91^ Additionally, caspase-1 drives pro-IL-1β and pro-IL-18 into their mature, active forms.^19,65,92^

In some cases, inflammasome stimulation leads to cytokine secretion without cell lysis. However, the mechanisms that regulate the phenomenon remain to be further unveiled. Research suggests that the Toll-like receptor adapter SARM may be involved in this process.^93^

### Non-canonical pathway

In non-canonical pyroptosis, human caspase-4/5 (caspase-11 in mice) is activated by the intimate binding of lipopolysaccharides (LPS) to their N-terminal CARDs.^94^ Upon activation, caspase-4/5/11 cleaves GSDMD, generating an N-terminus that is inserted into the membrane to form pores.^95^ However, unlike caspase-1, caspase-4/5/11 are unable to cleave pro-IL-1β/IL-18 precursors on their own, and IL-1β/IL-18 release requires engagement of the NLRP3/caspase-1 pathway in specific cellular contexts.^96^ The processing of GSDMD by caspase-4/5/11 elicits K^+^ efflux, which leads to NLRP3 inflammasome formation and pyroptotic cell death similar to the classical pathway.^19,97–99^ Unlike the classical pathway, in non-canonical pyroptosis, only cleavage of pro-IL-1β/IL-18 depends on caspase-1, while cleavage of GSDMD is performed by other activated inflammatory caspases. Pannexin-1 is another critical mediator of non-canonical caspase-11-induced pyroptosis.^100^ LPS exposure activates caspase-11, which cleaves pannexin-1, leading to ATP efflux that triggers P2X7-mediated pyroptosis. Notably, pannexin-1 deficient mice exhibit endotoxin shock resistance, indicating that K^+^ channels are selective modulators of non-canonical NLRP3 stimulation.

Additionally, the NLRP3 inflammasome regulates GSDME expression in human T cells. The NLRP3 inflammasome significantly impacts the activation of caspase-8, caspase-3, and GSDME cleavage, ultimately releasing the alarm signal IL-1α in a specific subset of T helper cells targeting *Candida albicans*. This process is triggered by calcium-licensed calpain maturation of pro-IL-1α after T cell receptor stimulation. The mechanism of GSDME pore formation in T cells for cytokine release depends on the NLRP3 inflammasome.^101^

### Caspase-3/8–mediated pathway

Previously thought to be inert to gasdermin activation, caspase-3, an apoptotic caspase, has been found to lead to chemotherapy-induced GSDME fragmentation in cells with abundant GSDME, resulting in the liberation of pyroptosis-inducing N-termini in tumors.^23,102,103^ This phenomenon has been observed in paclitaxel- and cisplatin-induced lung cancer cells and lobaplatin-induced colon cancer cells.^104,105^ Additionally, the Yersinia effector YopJ stimulates caspase-8 to cleave GSDMD by hindering TAK1 in mouse macrophages, further expanding our mechanistic understanding of pyroptosis.^24,25^

Caspase-8, another apoptosis-related caspase, has also been implicated in pyroptosis. In breast cancer cells, PD-L1 redirects TNF-induced death from apoptosis to pyroptosis. Under hypoxic conditions, activated STAT3 enters the nucleus along PD-L1, amplifying GSDMC transcription. TNF-α then promotes caspase-8-mediated cleavage of GSDMC, generating N-terminal fragments that perforate the membrane and elicit pyroptosis. Macrophage TNF-α-driven tumor cell pyroptotic death requires nuclear PD-L1, caspase-8, and GSDMC in vivo. Furthermore, certain chemotherapies induce caspase-8- and GSDMC-dependent pyroptotic death.^36^ Caspase-8 is often referred to as a molecular switch because it is critical in determining whether a cell’s fate is apoptosis, necroptosis, or pyroptosis.^106^

### Granzyme-mediated pathway

In 2020, Liu et al. demonstrated that chimeric antigen receptor (CAR) T-cells can quickly engage caspase-3 in target cells through granzyme B release, triggering the caspase-3/GSDME pyroptosis pathway and leading to widespread pyroptotic cell death.^37^ In addition, granzyme B directly fragments GSDME to stimulate pyroptosis, enhance anti-tumor immunity, and restrict tumor expansion.^26^ Natural killer and cytotoxic T cells have been observed to eliminate GSDMB^+^ cells via pyroptosis, with cytotoxicity resulting from granzyme A (GzmA)-mediated GSDMB fragmentation at Lys229/Lys244. Notably, non-aspartic gasdermin processing and pore formation by GzmA redefined our previous understanding and expanded the potential for pyroptosis induction beyond caspases.^27^ Different splicing variants of GSDMB have distinct functions, with the cleaved N-terminal fragments of only certain isoforms causing pyroptosis.^107^

## Biological functions in health

The large GSDM family is representative of the widespread phenomenon of pyroptosis in mammals, suggesting that pyroptosis is a vital mode of RCD.^108^ Scientists have investigated the role of pyroptosis in biological processes, and evidence suggests that pyroptosis likely plays a role in combating infections by eliminating intracellular pathogen replication sites and supporting downstream immune responses.

Inflammasomes are PRRs that recognize intracellular and extracellular pathogen ligands. Sensors of pyroptosis respond to components of bacteria and viruses, leading to the secretion of IL-18 and IL-1β through gasdermin-forming pores and plasma membrane rupture with the release of DAMPs, including HMGB1, ATP, and others. These cellular contents subsequently trigger several cellular events, including inflammation, proliferation, and differentiation.^22,47,109^ According to preliminary studies, IL-18 and IL-1β are critical for innate and adaptive immunity. NK and Th1 cells express IL18R on their surface and are highly sensitive to IL-18, which forms a positive loop with interferon-γ.^110^ Thus, pyroptosis is vital in the crosstalk between innate and adaptive immunity, inducing an immunostimulatory response against pathogens.^111^ As a crucial contributor to innate immunity, pyroptosis leads to the generation of pore-induced intracellular traps (PITs), i.e., structures that entrap previously intracellular microbes. PITs facilitate intracellular bacterial clearance by containing the pathogen and producing signals that promote recruitment and uptake by neutrophils.^80^ Besides, emerging research has suggested that pyroptosis exerts an antagonistic effect on endogenous danger signals, such as oxidative stress, in addition to triggering a microbe-mediated immune response.^112,113^

Aside from lytic cell death, gasdermins, the key executors of pyroptosis, participate in a wide range of physiological processes, including cell differentiation, tissue homeostasis, mitochondrial homeostasis, immune tolerance, and neutrophil extracellular trap (NET) formation.^114–116^ GSDMA3 was reported to play a vital role in normal hair follicle differentiation by participating in the Msx2/Foxn1/acidic hair keratin pathway.^117^ Li et al. reported that in osteoblasts, GSDMD underwent cleavage to generate non-lytic p20 products, which helped prevent bone loss and maintain bone homeostasis.^118^ Besides, GSDMD was identified as a key factor in the separation of bacteria from the epithelium in the colon owing to its role in goblet cell-mediated mucus layer formation. This biological function of GSDMD depends on its regulation of cortical F-actin disassembly in the process of mucin secretion.^114^ According to a recent study, the N-terminal domain of Gsdma3 was associated with Hsp90 and targeted mitochondria, thus regulating mitochondrial oxidative stress.^115^ In intestinal epithelial cells (IECs), GSDMD was cleaved by caspase-3/7 to produce a 13 kD N-terminal fragment upon exposure to dietary antigens. This fragment translocated to the nucleus and stimulated the expression of CIITA and MHCII molecules, which induced type 1 regulatory T (Tr1) cells and enabled immune tolerance of IECs to dietary antigens.^119^ Moreover, neutrophil proteases catalyze the proteolytic activation of GSDMD during NETosis, affecting nuclear expansion and protease activation via a feed-forward loop.^116^

## Regulatory function of pyroptosis in diseases

**Fig. 3:**
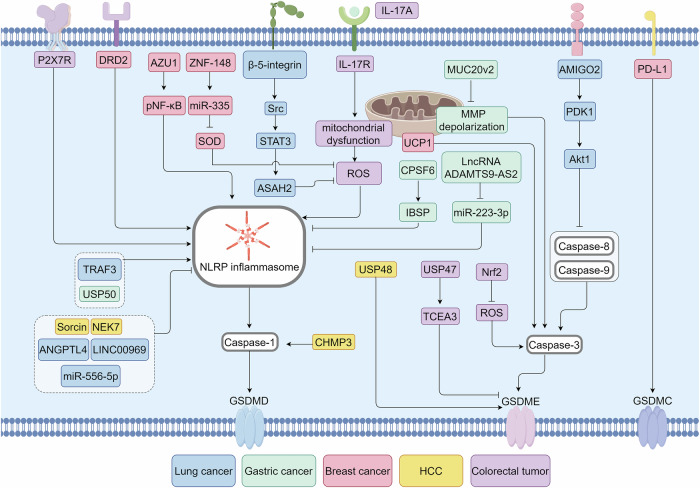
Regulatory mechanisms of pyroptosis in cancer. Over recent years, a series of molecules have been proven to target different sites of the pyroptosis pathway, thereby influencing the occurrence and progression of various cancers, including lung cancer, gastric cancer, breast cancer, HCC, and colorectal cancer. AZU1 Azurocidin 1, Akt protein kinase B, ASAH2 sphingolipid metabolic enzyme ceramidase, CPSF6 cleavage and polyadenylation factor 6, CHMP3 charged multivesicular body protein 3, DRD2 D2 dopamine receptor, IBSP integrin binding sialoprotein, MUC20v2 MUC20 variant 2, MMP mitochondrial membrane potential, P2X7R P2X7 receptor, PDK1 3-­phosphoinositide-­dependent kinase 1, Sorcin soluble resistance-related calcium-binding protein, TRAF3 tumor necrosis factor receptor-associated factor 3, TCEA3 transcription elongation factor a3, UCP uncoupling protein 1, ZNF Zinc finger protein 148. The figure was created by Figdraw

**Fig. 4:**
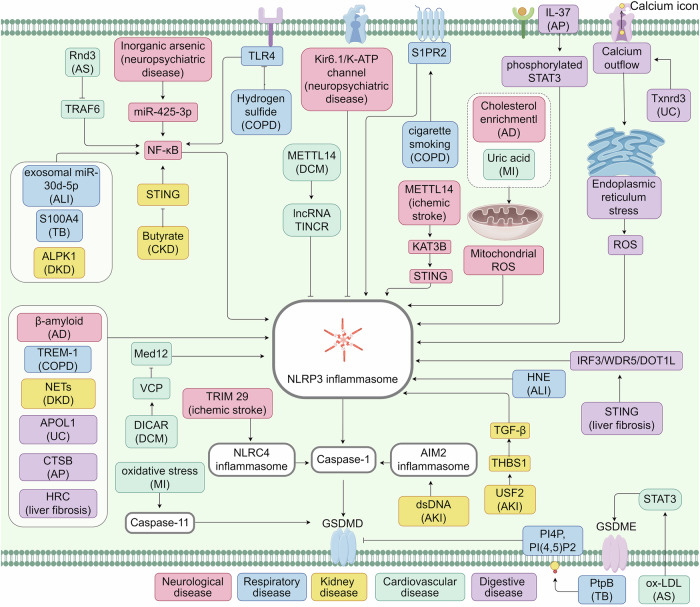
Regulatory mechanisms of pyroptotic cell death in non-cancer disease. Research on pyroptosis fosters the pathological process comprehension of systemic diseases and the underlying mechanisms involving numerous proteins, RNAs, and other molecules. ALPK1 alpha‐kinase1, CTSB cathepsin B, HRC histidine-rich calcium-binding protein, HNE 4-hydroxynonena, METTL14 methyltransferase-like 14, NETs neutrophil extracellular traps, PI(4;5)P2 phosphatidylinositol-(4;5)-bisphosphate, PI4P phosphatidylinositol-4-monophosphate, S1PR2 sphingosine-1-phosphate receptor 2, Txnrd3 thioredoxin reductase 3, TRIM29 tripartite motif containing 29. The figure was created by Figdraw

Pyroptosis facilitates the clearance of injured or infected cells, comprising a crucial defense against pathogen infection and DAMP stimulation. Persistent pyroptotic cell death can result in ion gradient dissipation, cellular content release, and exaggerated inflammatory responses, contributing to the pathophysiology of various diseases, including cancer. Two diagrams summarize the regulatory mechanisms of the pyroptotic pathway in cancerous and non-cancerous diseases (Figs. 3 and 4).

### Cancer

Pyroptosis plays a dual role in cancer, exhibiting context-dependent pro- or anti-tumor effects during tumorigenesis, because of its significant impact on inflammation and immunity.^120,121^ Its divergent functions in cancer are influenced by tumor characteristics, genetic background, host immunity status, and the specific pyroptotic effectors involved, emphasizing the complex interplay between pyroptosis, innate immunity, and the tumor microenvironment.^122–126^ Below, we summarize the involvement of pyroptosis in a range of cancers, covering lung, gastric, breast, hepatocellular carcinoma (HCC), and colorectal cancer.^127–131^

#### Lung cancer

Lung cancer is highly prevalent and a major factor contributing to death worldwide.^132^ Inflammasomes, the key initiators of the pyroptotic pathway, were highly expressed in various types of lung cancer. Several genes have been implicated in the regulation of inflammasome expression, most notably, *NLRP3*. In lung cancer cells, long non-coding RNA (lncRNA) LINC00969 promotes resistance to gefitinib via epigenetic inhibition of NLRP3. Mechanistically, LINC00969 binds to both METTL3 and EZH2, transcriptionally modifies the level of H3K27me3 in the region of NLRP3 promoter, and post-transcriptionally regulates the m6A level in NLRP3.^127^ Inhibition of tumor necrosis factor receptor-associated factor 3 (TRAF3) promotes the progression of lung adenocarcinoma (LUAD) cells and weakens the sensitivity of the cells to paclitaxel, partly through activation of caspase-1-dependent pyroptosis.^133^ In lung and pancreatic cancer models, β5-integrin upregulated the Src-STAT3-ASAH2 signaling axis, thus reducing ROS production to prevent chemotherapy-induced pyroptosis.^134^ ANGPTL4, a critical regulatory gene for lipid and glucose metabolism, was found to contribute to gefitinib resistance in non-small cell lung cancer (NSCLC) cells by regulating the NLRP3/ASC/caspase 8 pathway.^135^ Silencing lncRNA-XIST promotes NLRP3-mediated pyroptosis and NSCLC progression via activating the miR-335/SOD2/ROS pathway.^136^ Shi et al. downregulated miR-556-5p expression to increase cisplatin sensitivity in cisplatin-resistant NSCLC (CR-NSCLC) tissues. Mechanistically, the downregulation of miR-556-5p contributes to the expression of NLRP3, thereby provoking pyroptosis in cisplatin-treated CR-NSCLC cells.^137^ NLRP3-independent pyroptotic pathways influence the onset and progression of lung cancer. For example, AMIGO2 attenuated the (caspase-8 and caspase-9)/caspase-3 cascade by initiating the PDK1/Akt (T308) signal axis, attenuating GSDME-mediated pyroptosis, which reduces the innate sensitivity of NSCLC cells to cisplatin.^138^

#### Gastric cancer

Gastric cancer is among the primary causes of cancer-related mortality worldwide and is often discovered at an advanced stage when surgical intervention is no longer appropriate.^139^ MUC20 variant 2 (MUC20v2) protects gastric cancer cells from apoptosis and pyroptosis by maintaining mitochondrial calcium levels and membrane potential homeostasis, which enhances cell survival and chemoresistance.^128^ LncRNA ADAMTS9-AS2 is a tumor suppressor and sensitizes chemoresistant gastric cancer cells to cisplatin by upregulating the miR-223-3p/NLRP3 signaling axis.^140^ USP50 enhances bile acid-induced NLRP3-mediated pyroptosis of macrophages, releasing HMGB1 and leading to the genesis of gastric tumor cells through the PI3K/AKT and mitogen-activated protein kinase (MAPK)/extracellular signal-regulated kinase (ERK) pathways.^141^ Cleavage and polyadenylation factor 6 (CPSF6) and *Helicobacter pylori* also regulate pyroptosis in gastric cancer.^142,143^

#### Breast cancer

Breast cancer is one of the most prevalent malignancies worldwide.^144^ In the tumor microenvironment (TIM) of breast cancer, modulation of macrophages to the M1 phenotype by DRD2, a D2 dopamine receptor, leads to NLRP3 inflammasome assembly and subsequent pyroptosis.^145^ Azurocidin 1 (AZU1), a heparin‑binding protein, was reported to induce pyroptosis by initiating the pNF‑κB/NLRP3/caspase‑1/GSDMD signaling axis in triple-negative breast cancer (TNBC) in vitro.^146^ Zinc finger protein 148 (ZNF-148) was found to aggravate the progression of breast cancer through induction of miR-335/SOD2/ROS-mediated pyroptosis.^147^ Mitochondrial protein UCP1 was reported to inhibit TNBC progression by activating caspase-3-mediated pyroptosis.^129^ In addition, the PD-L1/PD-1 immune checkpoint has been found to engage in the pyroptosis pathway under specific circumstances when PD-L1 translocates to the nucleus, where it enhances GSDMC transcription. Subsequent treatment with TNF-α results in caspase-8-mediated cleavage of GSDMC, leading to pyroptosis and tumor necrosis in breast cancer.^36^

#### Hepatocellular carcinoma

HCC is one of the primary causes of cancer-related death, and its prevalence is anticipated to increase globally.^148^ A recent study found that soluble resistance-related calcium-binding protein (sorcin) impairs the assembly of the NLRP3 inflammasome, thus inhibiting cell pyroptosis and provoking HCC progression.^130^ A noteworthy association was found in HCC cells between the expression of GSDMD and NEK7. Moreover, NEK7 knockdown elevated pyroptosis-related markers NLRP3, caspase-1, and GSDMD, reducing HCC stimulation of hepatic stellate cells, hinting at NEK7’s considerable role in both tumor progression and cancer-stromal interactions in HCC.^149^ Additionally, USP48 stabilized GSDME by removing K48-linked ubiquitination at K120 and K189, thereby promoting pyroptotic death in liver cancer cells.^150^ Additionally, charged multivesicular body protein 3 (CHMP3) contributed to liver cancer via caspase-1-dependent pyroptotic cell death.^151^

#### Colorectal cancer

Owing to environmental degradation and population aging, colorectal cancer ranks third globally in terms of frequency of malignant tumors and has emerged as the world’s fourth most deadly cancer.^152^ IL-17A has been suspected of inducing mitochondrial dysfunction, intracellular ROS generation, and activation of the NLRP3/caspase-4/GSDMD pyroptotic pathway, consequently upregulating the secretion of inflammatory substances and recruiting infiltrating CD8^+^ T cells to colorectal tumors.^131^ The highly selective P2X7R antagonist, A438079, was observed to inhibit pyroptosis via the NLRP3/caspase-1 pathway, although it remains unclear whether this inhibition could ultimately prevent colorectal cancer progression.^153^ Moreover, a recent study revealed that Nrf2 inhibition increases the sensitivity of CRC cells to oxaliplatin by promoting pyroptosis and ferroptosis.^154^ USP47-driven deubiquitination and stabilization of transcription elongation factor a3 (TCEA3) suppressed pyroptosis of colorectal cancer cells promoted by chemotherapeutic doxorubicin.^155^

### Neurological disorders

Accumulating evidence points to pyroptosis as a critical factor in Alzheimer’s disease (AD), Parkinson’s disease (PD), ischemic stroke, multiple sclerosis (MS), traumatic brain injury, spinal cord injury, epilepsy, and neuropsychiatric and neurodevelopmental diseases.^156–162^ Below, we summarize the regulation of pyroptosis in a range of neurological diseases.

#### Alzheimer’s disease

AD is the most common neurodegenerative disease in the elderly and is characterized by cognitive decline. The major neuropathological features of AD include amyloid β (Aβ) plaques and neurofibrillary tangles (NFT), along with neuroinflammation and neuronal loss.^163^ Accumulating evidence indicates that the regulation of pyroptosis plays a role in the pathology of AD.^164^ Moonen et al. demonstrated differential activation of the pyroptotic pathway in a cell type-dependent manner in AD cases, implicating pyroptosis activation in neuronal death.^165^ Recent studies have revealed that β-amyloid could promote NLRP3-caspase-1-GSDMD signaling in neurons, leading to nerve injury in AD.^166,167^ β-amyloid protein aggregation around ASC fibrils could amplify the inflammatory response, leading to pyroptotic cell death.^168^ Other neuropathogenic proteins, including tau proteins, can induce NLRP3 inflammasome activation.^169^ In a vicious cycle, ASC released by microglia binds to β-amyloid, enhancing the formation of β-amyloid oligomers and aggregates, which can be attenuated by the anti-ASC antibody.^170^ Cholesterol overload in neuronal cells provokes pyroptosis via increasing Aβ‑induced oxidative stress in the mitochondria.^171^ Overall, neuropathogenic proteins can induce inflammasome formation and proinflammatory cytokine release, exacerbating AD pathology.

#### Parkinson’s disease

PD is a neurodegenerative disorder featuring misfolding of α-synuclein proteins and Lewy body formation in affected neurons.^172^ These neuropathogenic protein aggregations lead to the loss of dopaminergic neurons in the substantia nigra pars compacta, with symptoms of tremors and bradykinesia.^173^ The pathophysiology of PD correlates with the neuroinflammation theory and involves pyroptosis.^174^ A recent study reported that salsolinol induces the expression of pyroptosis-related proteins, such as NLRP3, ASC, and caspase-1, in SH-SY5Y cell lines and mouse models.^175^ In addition, elevated levels of cleaved caspase-1 and ASC have been identified in substantia nigra samples from patients with PD compared to healthy controls.^176^ Therefore, growing evidence from cellular, animal, and clinical studies supports a correlation between pyroptosis and PD progression.

#### Ischemic stroke

Known as a crippling central nervous system disease caused by arterial blockage with high mortality, ischemic stroke imposes a high burden on the economy and society. Increasing evidence suggests that inflammation promoted by inflammasomes and pyroptosis is essential for the pathophysiological process of ischemic stroke and is strongly linked to prognosis. METTL14 (methyltransferase-like 14) was found to activate the NLRP3 inflammasome/pyroptosis axis via KAT3B-STING signaling after oxygen-glucose deprivation/reperfusion (OGD/R), which simulates ischemic stroke in vitro.^177^ Wei et al. demonstrated that TRIM27 downregulated NLRP3 inflammasome-mediated pyroptosis through Akt/Nrf2/HO-1 signaling, ameliorating ischemic stroke.^178^ Likewise, TRIM29 (Tripartite Motif Containing 29) was reported to exert a negative regulatory effect on pyroptosis in ischemic stroke.^179^

#### Other neurological disorders

Associations have also been found between pyroptosis and some other neurological disorders, including MS, amyotrophic lateral sclerosis, and neuropsychiatric diseases. As a chronic inflammatory demyelination disorder, the development of MS is closely related to pyroptosis. Caspase-1-mediated GSDMD pyroptosis and inflammasome activation have been observed in oligodendrocytes (ODCs) and microglial cells in vitro and patients with MS.^180^ Activated caspase-3 with GSDMD immunopositivity has also been identified in macrophages/microglia within demyelinating lesions of patients with progressive MS and in experimental autoimmune encephalomyelitis (EAE) models.^181^ Motor neuron loss in the motor cortex is associated with increased inflammasome-triggered pyroptosis in microglia.^182^ NLRP3 inflammasome activation has also been observed in TDP43^A315T^ and SOD1 transgenic animals.^183,184^

Neuropsychiatric diseases, such as anxiety and depression, are characterized by cognitive and mental abnormalities. It has been demonstrated that in addition to hereditary variables, elevated inflammation plays a substantial role in many neuropsychiatric illnesses. Simon et al. reported that the monocytes of patients with major depressive disorder (MDD) exhibited signs of premature aging and inflammation, as well as a tendency toward pyroptotic cell death.^185^ A recent study illustrated that the Kir6.1/K-ATP channel in astrocytes negatively modulated astrocytic pyroptosis by preventing the assembly and activation of the NLRP3 inflammasome, which plays a vital role in the pathogenesis of depression.^186^ Furthermore, inorganic arsenic exposure may result in the development of generalized anxiety disorder (GAD) by downregulating the expression of miR-425–3p in the prefrontal cortex, which targets the NF-κB/NLRP3/caspase-1/GSDMD signaling axis and causes the release of IL-1β and IL-18.^187^

### Respiratory diseases

Research indicates that pyroptosis is critical for inflammatory diseases, with increasing evidence linking respiratory diseases to inflammation, bringing the role of pyroptosis in respiratory diseases to the forefront of research. Pyroptosis participates in the genesis of chronic obstructive pulmonary disease (COPD), acute lung injury (ALI), asthma, silicosis, pulmonary hypertension, cystic fibrosis, and pulmonary tuberculosis.^188–194^ In this section, we sum up the roles and corresponding mechanisms of pyroptosis in inflammatory respiratory diseases.

#### Chronic obstructive pulmonary disease (COPD)

COPD is a severe health issue and the third leading cause of inflammatory respiratory-related mortality globally.^195^ The most significant risk factor for COPD is cigarette smoke, which can draw inflammatory cells to the lung tissues and trigger the induction of a range of cytokines. Pyroptosis is irreversible when cigarette smoke induces tissue inflammation. For example, cigarette smoking can enhance the expression of sphingosine-1-phosphate receptor 2 (S1PR2) in human bronchial epithelial cells, thus triggering the NLRP3/ASC/caspase-1 pathway, leading to airway inflammation and injury.^189^ Likewise, cigarette smoke extract contributed to the pyroptosis of human bronchial epithelial cells via the ROS/NLRP3/caspase-1 axis.^196^ Meanwhile, hydrogen sulfide was reported to alleviate pyroptosis and lung injury in a model of smoking-induced COPD by downregulating the TLR4/NF-κB signaling pathway.^197^ Furthermore, Wang et al. proved that TREM-1 could activate NLRP3-mediated pyroptosis, thereby aggravating the injury and inflammation that COPD caused to lung tissues.^198^ Additional studies have revealed that factors, including lncRNA GAS5, TRPV4, and WSPM2.5, aggravated COPD by activating pyroptotic cell death.^199–201^ Inhibition of pyroptosis by exosomes from adipose-derived stem cells and the Nrf2/HO-1 signaling axis might alleviate COPD.^202,203^

#### Acute lung injury (ALI)

ALI is a lethal disease characterized by cytokine storms, leukocyte infiltration, and diffuse alveolar injury.^204^ Polymorphonuclear neutrophils (PMNs) are essential to inducing sepsis-related ALI. Specifically, exosomal miR-30d-5p from PMNs facilitated sepsis-related ALI by inducing M1 macrophage polarization and activating NF-κB to prime macrophage pyroptosis.^205^ A recent study revealed that pyroptosis contributed to ALI caused by cecal ligation and puncture (CLP), and aldehyde dehydrogenase 2 (ALDH2) acted as a buffer by limiting pyroptosis.^206^ A lipid peroxidation product, 4-hydroxynonenal (HNE), was found to attenuate NLRP3 inflammasome-mediated pyroptosis and downstream IL-1β release, independent of the Nrf2 and NF-κB signaling pathways, contributing to sepsis-related ALI.^207^ Furthermore, many other substances had a regulatory effect on pyroptosis, thus affecting the development of ALI induced by sepsis and other causes.^208–212^

#### Asthma

Asthma is a chronic respiratory inflammatory disease featuring reversible airflow limitation and airway hyperresponsiveness.^213^ Increasing evidence has shown the critical role of pyroptosis in asthmatic airway inflammation and injury. In an ovalbumin-induced asthmatic mouse model, GSDMD silencing significantly reduced Th17 and Th2 inflammatory responses as well as M2 macrophage polarization, all of which are involved in airway remodeling and inflammation.^214^ Dectin-1 activation in asthma provoked caspase-11/4-mediated macrophage pyroptosis, thereby stimulating the secretion of chemokines and aggravating airway neutrophil inflammation.^215^ MUC1 suppressed NLRP3 inflammasome-mediated pyroptosis via blocking the TLR4/ MyD88/NF-κB pathway, subsequently alleviating neutrophil airway inflammation in asthma.^216^

#### Silicosis

Attributed to long-term inhalation of crystalline silica (CS) particles, silicosis is a severe lung disease marked by irreversible pulmonary fibrosis.^217^ Emerging evidence has indicated that CS-induced lung injury was closely associated with the activation of pyroptosis. Meiyue Song et al found that macrophages in silicosis lung tissue underwent GSDMD-induced pyroptosis, mediated by both canonical and non-canonical signaling pathways.^218^ Another mechanistic study elucidated that silica exposure triggered the dysfunction of the P2X7 ion channel, causing intracellular K^+^ efflux and the formation of the NLRP3 inflammasome, which cooperated with LPS-primed activities to provoke macrophage pyroptosis and pulmonary inflammation.^219^ Furthermore, CS particles induced damaged mitochondria to release DAMPs, which initiated downstream NLRP3 inflammasome-mediated pyroptosis to promote pulmonary fibrosis.^191^

#### Pulmonary tuberculosis

Caused by *Mycobacterium tuberculosis* (Mtb), tuberculosis (TB) is a highly contagious illness that continues to be the most common infectious agent-related cause of mortality.^220^ Accumulating evidence has supported pyroptosis as a critical factor in the development of TB. Mycobacterial EST12 binds to the receptor for activated C kinase 1 (RACK1) in macrophages to assemble a complex that engages the deubiquitinase UCHL5 to facilitate the K48-linked deubiquitination of NLRP3, consequently promoting the NLRP3-caspase-1/11-IL-1β immune pathway.^221^ PtpB, an Mtb phospholipid phosphatase, was recently reported to suppress the host inflammasome-pyroptosis pathway. Mechanistically, PtpB dephosphorylates phosphatidylinositol-4-monophosphate (PI4P) and phosphatidylinositol-(4,5)-bisphosphate [PI(4,5)P2] in the host cell membrane, thereby interfering with the membrane localization of cleaved GSDMD and preventing macrophage pyroptosis.^222^ In addition to Mtb, host molecules regulate pyroptosis, driving TB disease progression. According to a recent study, S100A4 provoked the pyroptosis of THP-1 macrophages induced by BCG infection via activation of the NF-κB/NLRP3/caspase-1/GSDMD signaling pathways.^194^ Moreover, researchers have recently discovered many other factors regulating pyroptosis during pulmonary tuberculosis.^223–228^

### Kidney disease

Pyroptosis participates in the initiation and progression of kidney diseases, as most share characteristics such as inflammation and parenchymal cell death. Several studies have shown regulation of pyroptosis in the pathogenesis of kidney diseases, such as acute kidney injury (AKI), chronic kidney disease (CKD), diabetic kidney disease (DKD), lupus nephritis, and kidney allograft transplantation.^229–233^ This section reviews studies implicating pyroptosis in kidney disease models.

#### Acute kidney injury

Acute kidney injury (AKI) is a prevalent clinical complication with high worldwide morbidity and mortality rates.^234^ Increasing evidence suggests that pyroptosis significantly contributes to the pathogenesis of AKI. USF2 upregulated THBS1 to activate the TGF-β/NLRP3 signaling pathway, provoking pyroptosis and further aggravating sepsis-induced AKI.^235^ According to a recent study, dsDNA-induced AIM2 pyroptosis rapidly removes macrophages, which unexpectedly halts aberrant inflammation during AKI triggered by rhabdomyolysis.^236^ Baatarjav et al. revealed the pivotal role of GSDME-mediated pyroptosis in AKI development. The cleavage of GSDME by caspase-3 is responsible for forming membrane pores and cell lysis, exacerbating inflammation and renal tubular damage.^229^ Furthermore, recent investigations have identified numerous proteins, RNAs, and other molecules that implicate pyroptosis as a driver of AKI pathogenesis.^237–241^

#### Chronic kidney disease

CKD is an irreversible, progressive illness that massively affects health outcomes.^242^ Recent research has indicated that pyroptosis contributes to CKD progression. Butyrate plays a renoprotective role, alleviating renal fibrosis in CKD. Mechanistically, STING/NF-κB/p65 pathway downregulation attenuated NLRP3-mediated pyroptosis.^243^ GSDME was found to contribute to renal tubulointerstitial fibrosis and renal dysfunction induced by ureteral obstruction and 5/6 nephrectomy via pyroptotic cell death.^244^ In addition, a new study illustrated the collaboration of GSDMD and GSDME in the transition of AKI to CKD.^242^

#### Diabetic kidney disease

DKD is the main cause of end-stage renal disease, with a dramatically increased prevalence worldwide over the past decades.^245^ Recent studies have confirmed that pyroptotic death is essential in the development of DKD. During DKD, caspase-11/4 and GSDMD-mediated pyroptosis are activated, contributing to podocyte loss.^246^ A recent study demonstrated that alpha‐kinase1 (ALPK1) was activated by hyperglycemia and caused phosphorylation of NF-κB in renal tubular epithelial cells in DKD. Subsequently, the canonical caspase-1-GSDMD pyroptosis pathway is induced, contributing to tubular injury and interstitial inflammation.^231^ NETs have also been reported to induce glomerular endothelial cell (GEC) pyroptosis, mediated by charge, further inducing the development of DKD.^247^ Over the past few years, other molecules and substances, such as circRNA, LncRNA, and lysophosphatidic acid have also been reported to target the regulation of pyroptosis in DKD.^248–252^

### Cardiovascular diseases

Accumulating evidence has demonstrated that the regulation of pyroptosis influences the pathogenesis of cardiovascular diseases (CVD). Pyroptosis induces the amplification of inflammatory responses and accelerates the occurrence of cardiovascular diseases such as atherosclerosis, myocardial infarction (MI), arrhythmia, and cardiac hypertrophy.^253–255^

#### Atherosclerosis

Arteriosclerosis is a chronic inflammatory process featuring lipid deposition, plaque build-up, and endarterium thickening.^256^ As the primary etiological factor for cardiovascular and cerebrovascular disorders, atherosclerosis has become a significant global cause of death and disability.^257^ Emerging studies have suggested that pyroptosis is critical for the development of atherosclerosis. The lncRNA NEAT1 positively regulates the expression of NLRP3 at the transcription level, initiating pyroptosis of vascular endothelial cells. In contrast, exercise attenuates the function of NEAT1, impeding atherosclerosis plaque formation.^258^ Rnd3 was reported to downregulate the TRAF6/NF-κB/NLRP3 signaling pathway via regulation of TRAF ubiquitination, thus impairing endothelial pyroptosis in atherosclerosis.^259^ During atherosclerosis, STAT3 in macrophages is activated by ox-LDL or inflammatory cytokines and upregulates GSDME transcription, which increases caspase-3 activity and contributes to the transition from apoptosis to pyroptosis.^260^ Additionally, many other molecules participate in atherosclerosis by regulating the pyroptosis of endothelial cells or macrophages, including uric acid, homocysteine, nicotine, IQGAP1, and hormones.^261–266^

#### Myocardial infarction

Ischemic heart disease, particularly MI, remains the leading cause of death worldwide.^267^ Directly restoring blood flow in the ischemic region immediately is the most effective method for rescuing dying cardiomyocytes. Reperfusion, however, is a double-edged sword; while it can save ischemic myocardia, it also risks inflicting further damage, a condition known as myocardial ischemia/reperfusion (MI/R) injury.^268^ Emerging studies have attempted to uncover the pathophysiological role of pyroptosis in MI and MI/R injury. Uric acid was reported to exacerbate MI/R injury via upregulation of the ROS/ NLRP3 pyroptosis pathway.^269^ Oxytocin (OT) can exert protective effects against myocardial I/R injury with hyperglycemia via regulation of the AMPK/NLRP3 signaling pathway and pyroptosis.^270^ GSDMD-induced pyroptosis plays a critical role during MI/R injury, and the caspase-11/GSDMD pathway mediated by oxidative stress may be essential for the process.^271^ Furthermore, over the past few years, several additional mechanisms have been found to contribute to the pathogenesis of MI, such as circHMGA2, HIF-1α/TUG1/FUS, and lncRNA H19.^254,272–276^

#### Diabetic cardiomyopathy

Diabetic cardiomyopathy (DCM) is one of the most detrimental consequences of type 2 diabetes. DCM involves abnormal structural remodeling and aberrant cardiac function.^277^ Recent studies have widely reported the essential role of pyroptosis regulation in cardiomyopathy, particularly in DCM. Meng et al. discovered that METTL14 attenuates pyroptosis and DCM progression via m6A methylation of lncRNA TINCR by downregulating NLRP3 expression.^278^ Critically, CD38 deficiency alleviates type 2 diabetes-induced DCM by reducing pyroptosis and apoptosis in vitro and in vivo via activation of the NAD^+^/Sirt3/FOXO3a signaling pathways.^279^ CircRNA DICAR was identified as a novel endogenous regulator for DCM, with the potential to protect against cardiomyocyte pyroptosis via DICAR-VCP-Med12 degradation.^280^ What’s more, many other molecules in the human body exert regulatory effects on pyroptosis involved in DCM disease progression, such as folic acid, ghrelin, miR-21-3p, microRNA-223-3p, and lncRNA MIAT.^281–285^

#### Arrhythmia

Among the arrhythmia types, atrial fibrillation has been most frequently reported to be associated with cell pyroptosis. Atrial fibrillation is a prevalent arrhythmia in clinical practice, occurring in 1-2% of people worldwide, and has been linked to an increased risk of heart failure and hospitalization.^286^ Luo et al. discovered that *Akkermansia muciniphila* (*A.muciniphila*) protected rats against cold-related atrial fibrillation. Mechanically, cold exposure decreased the abundance of *A. muciniphila*, further causing augmented TMAO in plasma and TMAO-mediated atrial pyroptosis, which resulted in atrial structural remodeling and atrial fibrillation.^287^ According to a new study, the extracellular vesicles from adipose tissue-derived mesenchymal stem cells (AMSCs) carrying LncRNA XIST attenuated myocardial cell pyroptosis in atrial fibrillation via blocking miR-214-3p-mediated Arl2 inhibition.^288^

#### Cardiac hypertrophy

Pathological hypertrophy of the myocardium is one of the largest contributors to heart failure and has been associated with pyroptosis in recent studies.^289^ In a model of doxorubicin (Dox)-induced non-ischemic dilated cardiomyopathy, Dox increased the expression of NOX1 and NOX4 and triggered mitochondrial fission via dynamin-related protein 1 (Drp1) activation, resulting in caspase-1-dependent pyroptosis in cardiomyocytes.^290^ Moreover, TRPA1 deficiency aggravated dilated cardiomyopathy through augmenting S100A8 expression to induce pyroptosis in M1 macrophages.^291^ Yue et al also reported that NLRP3/caspase-1-dependent pyroptosis has a pathological role in myocardial hypertrophy.^292^ In a model of pressure-overload cardiac hypertrophy, NLRP3 deficiency exerted cardioprotective effects via activation of TAK1.^293^ Another study found that Sema4D contributed to pathological myocardial hypertrophy via the MAPK/NF-κB/NLRP3 pathway.^294^

#### Drug-induced cardiac injuries

Antineoplastics, antipsychotics, and other drugs can cause toxicity that adversely influences the heart and may result in cardiomyopathies, known as drug-induced cardiac injuries.^295^ Increasingly, studies have found an association between drug-induced cardiac injury and pyroptosis. Dox enhanced the expression of Bnip3, which activated caspase-3 to induce GSDME-mediated pyroptosis in cardiomyocytes.^296^ Additionally, mir-34a-5p selectively attenuated the expression of Sirt3 and then augmented autophagy and mitoROS generation, ultimately exacerbating Dox-induced myocardial cell pyroptosis.^297^ Antipsychotics competitively bonded to CB1R, which was then internalized and directly interacted with NLRP3 inflammasome through amino acid residues 177–209, mediating stabilization of the inflammasome and activation of cardiomyocyte pyroptosis.^298^

### Digestive diseases

The pyroptotic pathway has emerged as an indispensable component of digestive disorders, including gastric ulcer, ulcerative colitis (UC), acute pancreatitis (AP), and hepatic fibrosis.^299–302^ In this section, we summarize the role of pyroptosis in the pathological processes of digestive diseases.

#### Ulcerative colitis

UC is an aggressive, chronic inflammatory bowel disease accompanied by a high risk of bowel cancer.^303^ Accumulating evidence supports pyroptosis as a critical factor in the development of UC. Inhibition of the C3a/C3aR axis in the early stages of UC induces poor prognosis of UC by promoting cell pyroptosis. However, stimulation with a C3aR inhibitor in the later stages of UC alleviated the symptoms of UC by suppressing pyroptosis.^304^ Overexpression of Txnrd3 induced intracellular calcium outflow, endoplasmic reticulum stress, and ROS accumulation, ultimately leading to pyroptosis and necrosis of colon cancer cells.^305^ APOL1 overexpression induces the NLRP3/caspase1/GSDMD pyroptosis pathway and promotes the release of the chemokine CXCL1, further aggravating UC.^306^ Besides, lncRNA MEG3, miR-141-3p, and SLC6A1 have been found to influence the progression of UC via the regulation of pyroptosis.^307–309^

#### Acute pancreatitis

AP is an urgent and severe abdominal disease that can have both local and systemic consequences.^310^ Growing evidence has suggested a close correlation between the inflammasome and its downstream effectors and AP.^311^ Cathepsin B (CTSB) was reported to induce NLRP3/caspase-1-mediated cell pyroptosis, aggravating AP.^312^ A recent study uncovered that IL-37 impaired phosphorylated STAT3 and protected against pyroptosis of injured acinar cells in AP.^313^ Inhibition of TRAF6 was found to attenuate pancreatic injury in hyperlipidemic AP, along with relief of the inflammatory response via pyroptotic cell death.^314^ High-density lipoprotein (HDL) was reported to relieve oxidative stress and suppress acinar cell pyroptosis, reducing the severity of AP.^315^ Furthermore, additional factors upregulate cell pyroptosis and promote the progression of AP, such as circHIPK3, endogenous tRNA-derived small RNA, USP25, endoplasmic reticulum stress, and exosomal miR-155-5p.^316–320^

#### Liver fibrosis

Liver fibrosis is a chronic liver injury with the potential to progress to cirrhosis and correlates with an elevated risk of HCC over time.^321^ Accumulating evidence has illustrated that pyroptosis plays a vital role in the development of liver fibrosis. According to a recent study, NLRP3 overactivation resulted in hepatocyte pyroptosis and the release of inflammasome particles into the extracellular space, engendering hepatic stellate cell activation and liver fibrosis.^322^ STING was found to mediate hepatocyte pyroptosis through epigenetic activation of the IRF3/WDR5/DOT1L transcription activator complex, contributing to liver fibrosis.^323^ Hepatic histidine-rich calcium-binding protein (HRC) induced paracrine activation of hepatic stem cells (HSCs) by triggering hepatocyte pyroptosis via the NLRP3/caspase-1/HMGB1 signaling axis in liver fibrosis, promoting fibrogenesis.^324^ In addition, angiotensin II, S100A8, and the METTL3/MALAT1/PTBP1/USP8/TAK1 axis were proven to aggravate liver fibrosis. Concurrently, growth arrest-specific 5 (GAS5) and exosomes produced from bone marrow mesenchymal stem cells exerted an inhibitory effect on liver fibrosis.^325–329^

## Therapeutic strategy regulating pyroptosis

### Cancer

It is widely accepted that pyroptotic cell death is implicated in tumor suppression. Generally, activation of the pyroptosis signaling pathway augments anti-tumor immunity, whereas inhibition of the pyroptosis signaling pathway facilitates tumor growth and metastasis. In this section, we review the current literature on molecules that induce or enhance pyroptosis. Notably, some of these molecules have been used in cancer treatment for years, and their mechanisms have previously been solely attributed to the induction of apoptosis.

#### Therapeutic strategies targeting inflammasome

Inflammasomes are involved in various stages of tumor development and provide tumorigenic and tumor-suppressive functions.^5,330,331^ Owing to the role of pyroptosis in tumor initiation, targeting inflammasome activation is an encouraging therapeutic policy for cancer treatment.

Molecules that target the NLRP3 inflammasome have received extensive attention. Studies have shown that in tumor cells undergoing Snail-mediated epithelial-mesenchymal transition (EMT), the activity of NLRP3 inflammasomes in tumor-associated macrophages (TAMs) is diminished in response to chemotherapeutic agents. This suppression occurs via the transfer of exosomal miR-21, downregulating PTEN and BRCC3, leading to phosphorylation and lysine-63 ubiquitination of NLRP3. This process effectively prevents the assembly of the NLRP3 inflammasome, resulting in reduced chemotherapy efficacy.^332^ Therefore, triggering NLRP3-mediated pyroptosis of cells in the tumor microenvironment possesses treatment potential.

Yuan et al. identified cucurbitacin B, derived from muskmelon pedicel, as a natural bioactive compound demonstrating potent anti-tumor effects in lung carcinoma. Cucurbitacin B directly combined with TLR4, triggering the activation of the NLRP3 inflammasome. Subsequently, this activation led to the cleavage of gasdermin D into its N- and C-terminal domains, facilitating the induction of pyroptosis.^333^ The therapeutic potential of alpine pine flavones (AIF) against HCC has been investigated and demonstrated suppression of proliferation, migration, and invasion in Huh7 and SMMC7721 cell lines, possibly through the excitement of NLRP3 inflammasome assembly, which has also been verified by in vivo findings.^334^ Metformin, recognized for its glucose-lowering effects, also exhibits antineoplastic effects in HCC by triggering apoptosis and pyroptosis. This activity is mediated by the enhancement of FOXO3 expression by metformin, which subsequently increases NLRP3 transcription and facilitates pyroptosis.^335^ Another camptothecin anticancer drug, FL118, can also inhibit the progression and metastasis of colorectal cancer by inducing NLRP3-ASC-Caspase-1 mediated pyroptosis.^336^ A significant correlation was noted between the expression of GSDMD and the presence of NEK7. Silencing NEK7 led to the upregulation of markers associated with pyroptosis, including NLRP3, caspase-1, and GSDMD, thereby diminishing the activation of stellate cells in HCC, suggesting that NEK7 modulates tumor progression and the interaction between cancer and stromal cells in HCC.^149^ Besides, the administration of IL-17A triggers pyroptosis via the ROS/NLRP3/caspase-4/GSDMD axis, enhancing the release of proinflammatory cytokines and resulting in increased infiltration of CD8^+^ T cells within tumor tissues.^131^ Zhao et al. discovered that enhanced expression of circAR-3 escalates cell proliferation and inflammation in prostate cancer (PCa), whereas its suppression exerted contrary effects. This process is facilitated by the acetylation of NLRP3 by KAT2B, furthering the subcellular localization and assembly of the NLRP3 inflammasome complex. This finding suggests that inhibiting NLRP3 acetylation or inflammasome assembly could be a viable strategy for halting the advancement of PCa.^337^ Yan et al. demonstrated that cisplatin induces the NLRP3/caspase-1/GSDMD pyroptosis pathway through the elevation of long non-coding RNA (lncRNA) maternally expressed gene 3 (MEG3) in TNBC, contributing to its anti-tumor activity. This finding offers a potential new approach for therapeutic intervention in TNBC.^338^ However, because of the pyroptosis activation effect of cisplatin, there can be severe side effects including acute kidney injury and hearing loss.^339,340^ To alleviate the side effects induced by cisplatin, animal and cell tests of two potential therapeutic oral anticancer drugs, AZD5438 and dabrafenib, a phase-2 clinical trial protein kinase CDK2 inhibitor and a US Food and Drug Administration-approved drug BRAF inhibitor, respectively, were conducted with the promising result of reduced cell death.^341^ Therefore, it exemplifies that the side effect of pyroptosis-targeted drugs can be improved when they are combined with other targeted drugs. Conversely, coenzyme Q0, a derivative quinone from *Antrodia camphorate*, exerted anticancer activity by counteracting NLRP3-mediated inflammation. Coenzyme Q0 suppresses HIF-1α expression and inhibits the NLRP3 inflammasome, as well as ASC/caspase-1 expression, resulting in the downregulation of IL-1β and IL-18 expression in MDA-MB-231 and 468 cells and inhibition of EMT/metastasis of human TNBC and HNSCC cells.^342,343^ This opposite result implies that in different cancers and different periods of tumor progression, pyroptosis may play different roles, and the treatment strategy should vary accordingly.

Extracts from natural crops have been shown to regulate pyroptosis as well. Radix *Sophorae tonkinensis* oxymatrine extract possesses anticancer properties. Oxymatrine has anticancer effects against CRC by suppressing LRPPRC, promoting mitophagy, and inhibiting the NLRP3 inflammasome in CRC cell xenografts and liver metastasis models.^344^ A clinical trial of oxymatrine for the treatment of severe plaque psoriasis found only minor adverse effects, and a clinical trial for CRC is expected.^345^ Peimine (PM), derived from *Fritillaria*, was found to mitigate inflammasome activity by reducing endoplasmic reticulum (ER) stress and decreasing the levels of various proteins within the NF-κB and mitogen-activated protein kinase (MAPKs) pathways, limiting the proliferation of breast cancer cells.^346^

Moreover, applying nanoparticles to regulate NLRP3-mediated pyroptosis is another promising therapeutic strategy. Guo et al. developed novel VB12-tethered nano micelles by enhancing sericin with poly(benzyl-l-glutamate) (PBLG) and encapsulating the near-infrared dye IR780. Under near-infrared light exposure, these specialized nano micelles disrupt ATP synthase function, leading to mitochondrial impairment and subsequent ROS generation. This sequence of events triggered the NLRP3/caspase-1/GSDMD pathway, ultimately resulting in the maturation of dendritic cells.^347^

Targeting the AIM2 inflammasome also affects cancer development. Curcumin, the principal active compound in turmeric, triggers caspase-1/GSDMD-dependent pyroptosis in leukemia cells by boosting the expression of the IFI16, AIM2, and NLRC4 inflammasomes through the stimulation of ISG3 transcription factor activity.^348^ Li et al. showed that dihydroartemisinin (DHA) activates the AIM2/caspase-3/GSDME axis, initiating pyroptosis in breast cancer cells.^349^ Moreover, elevated CCL19 expression markedly suppresses the proliferation of gastric cancer cells and tumor progression, both in vitro and in vivo, by enhancing the CCR7/AIM2 pathway. These findings present an encouraging therapeutic tactic for the treatment of gastric cancer in cases of combination with chemical materials.^350^ Xu et al. introduced a virus-mimicking particle self-assembled from elongated DNA structures produced via rolling circle amplification (RCA) enveloped in cationic liposomes. This construction initiates the activation of the AIM2 inflammasome, leading to gasdermin D-mediated pyroptosis and enhanced anti-tumor immune responses.^351^ Furthermore, the development of biodegradable Ca^2+^ nanomodulators (CaNMs) for pyroptosis-mediated cancer immunotherapy has shown promise, as they cause mitochondrial Ca^2+^ overload and promote ROS generation, cytochrome C release, and ultimately, the caspase-3/GSDME-dependent pyroptotic pathway.^352^ Furthermore, in an anaplastic thyroid cancer clinical trial, apatinib upregulates caspase‐1 and melittin activates AIM2 inducing caspase‐3–GSDMD and caspase‐1–GSDME pyroptosis.^353^

#### Therapeutic strategies targeting caspases

Caspases are critical effectors of pyroptotic cell death and, therefore, have received much attention as a potentially novel strategy for tumor therapy.^104,105,354^ Compounds targeting caspase-3 have been favored. For instance, triptolide treatment inhibits the expression of mitochondrial hexokinase and c-Myc II in cancer cells, activating the BAD/BAX caspase-3 cascade and ultimately leading to GSDME-dependent pyroptosis.^355^ Another small compound, gambogic acid, can also induce caspase-3/GSDME pyroptosis, simultaneously enhancing cancer immunotherapy.^356^ Miltirone, a natural substance with anticancer activity, simultaneously prompts the proteolytic cleavage of GSDME and caspase-3, thereby inhibiting the viability of HepG2 or Hepa1-6 cells. Mechanistically, miltirone elicits intracellular ROS accumulation, inhibiting the phosphorylation of mitogen-activated and extracellular signal-regulated kinase (MEK) and extracellular regulated protein kinase 1/2 (ERK1/2) and inducing GSDME-dependent pyroptosis. ROS/ERK1/2-BAX-caspase-9-caspase-3-GSDME has been confirmed as the central signaling axis in the regulation of pyroptosis.^357^ Similarly, tetraarsenic hexoxide can also induce the generation of mitochondrial ROS leading to the caspase-3/GSDME-mediated pyroptosis in cancer cells.^358^ Nitidine chloride (NC), a benzophenanthridine alkaloid extracted from the Chinese medicinal herb *Zanthoxylum nitidum*, inhibits the phosphorylation of PI3K and Akt, thus increasing caspase-3/GSDME-mediated pyroptosis in lung cancer cells, indicating that NC is a prospective therapeutic agent for the treatment of lung cancer.^359^ Curaxin CBL0137, designed to modulate p53 and nuclear factor-κB, has demonstrated the capacity to deactivate the chromatin remodeling complex. This deactivation facilitates chromatin transcription, thereby reducing the transcription of antioxidant genes and promoting oxidative stress, leading to elevated ROS levels. Subsequently, mitochondrial ROS recruit BAX to the mitochondrial membrane, releasing cytochrome c and subsequently activating caspase-3. This cascade prompts caspase-3/GSDME-mediated pyroptosis in ovarian cancer cells.^360^ Germacrone, a sesquiterpene component obtained from the essential oil of Ezhu, demonstrated anticancer properties by inducing pyroptosis in liver cancer. This effect is mediated through the proteolytic cleavage of caspase 3, accompanied by the cleavage of GSDME, and is notably associated with elevated ROS production.^361^ Besides, *cordyceps militaris* extract can also induce caspase-3/PARP/GSDME pyroptosis.^362^ Although these molecules have not been evaluated in clinical trials, their pyroptosis-targeting effects can harbor great potential for disease treatment.

Xie et al. developed inhaled poly(lactic-co-glycolic acid) (PLGA) porous microspheres loaded with doxorubicin (DOX) and decitabine (DAC), which resulted in elevated expression of cleaved caspase-3 and promotion of cell pyroptosis.^363^ Additionally, two self-assembling protein nanotoxins, T22-DITOX-H6 and T22-PE24-H6, were designed to target chemokine receptor 4 (CXCR4) in head and neck squamous cell carcinoma (HNSCC) cells and to promote caspase-3/GSDME-mediated pyroptosis.^364^ Hu et al. reported a delivery strategy using arsenic trioxide nanoparticles (As2O3-NPs) for HCC treatment. In this approach, caspase-3 is activated to cleave GSDME and release its free N-terminal domain, triggering pyroptosis. Compared with free As2O3 and the control, As2O3-NPs showed better inhibition and induced more pronounced pyroptosis, increasing GSDME-N expression in Huh7 cells.^365^ These studies show that treatment in the form of nanoparticles may provide an ideal effect; however, more evidence and clinical trials are needed before they can be clinically applied because of potential side effects induced by the components and concerns of biocompatibility.

Targeting caspase-1/GSDMD-induced pyroptosis requires further extensive research. Simvastatin, traditionally used to manage hyperlipidemia, has garnered attention for its novel anticancer properties. Recent studies have revealed its ability to generate intracellular ROS in colon cancer cells, subsequently activating caspase-1 and initiating caspase-1-dependent pyroptosis.^366^ However, the ROS-generating effect of simvastatin was evaluated in 2016, with the results showing nonsignificant differences with the placebo group.^367^ Therefore, further evidence is required for the treatment outcome of colon cancer in vivo. Likewise, Chen et al. reported a similar phenomenon for secoisolariciresinol diglucoside in colorectal cancer cells and saikosaponin D in lung cancer cells.^368,369^ Additionally, a combination of drugs may enhance the ability to target caspase-1. For example, combined with ruthenium (II) polypyridyl complex Δ-Ru1, taxol improved caspase-1/GSDMD induced pyroptosis in Taxol-resistant cancer cells.^370^

Furthermore, other caspases, such as caspase-8 and caspase-9, have been explored for their interactions with pyroptosis in tumor cells, illustrating a diverse range of potential therapeutic approaches.^349,371–373^ Moreover, a mechanism of sorafenib therapy against HCC tumors through inducing macrophage (MΦ) pyroptosis and triggering an NK-cell response was explored. Pyroptosis in MΦ via upregulating caspase-1 activity caused the release of proinflammatory cytokines, enhancing the proliferation and activation of NK cells. Subsequently, tumor cells undergo apoptosis due to NK cell cytotoxicity, degranulation, and release of perforin.^374^ Notably, a phase III clinical trial of sorafenib in the treatment for HCC produced outcomes of unsatisfactory response and survival benefit, which was less significant than that of tislelizumab. However, the potential of combinations with other components is receiving extensive attention.^375–377^

These developments highlight the potential of targeting caspases and other related pathways as effective strategies for promoting pyroptosis in tumor therapy.

#### Therapeutic strategies targeting GSDM

Gasdermin is a potential target for anti-tumor therapeutic strategies because it is a critical effector of the pyroptosis axis. Endosomal sorting complexes necessary for transport (ESCRT) III-dependent cell membrane repair have been shown to effectively reduce pyroptosis in tumor cells by repairing and removing GSDM pores. Zhao et al. used a biodegradable, sustained-release calcium chelator based on nanoparticles to design a hydrogel-based delivery system aimed at blocking ESCRT III-dependent membrane repair, with the potential for improved immunotherapy efficacy.^378^ Another approach involves constructing a recombinant adeno-associated virus (rAAV) system to produce and deliver GSDMNT into tumor cells. Utilizing the Sf9/rBac system, a mammal-specific promoter for packaging rAAV-GSDMDNT, and employing the Cre/lox system to recover and express double-floxed inverted GSDMNT, the system stimulated immune responses by triggering pyroptosis and temporarily opening the blood-brain barrier (BBB).^379^ These works showed that innovative delivery approaches may improve targeting efficacy and safety.

In addition, the PD-L1/PD-1 immune checkpoint has been found to engage the pyroptosis pathway. Under specific circumstances, PD-L1 translocates to the nucleus, where it enhances GSDMC transcription. Subsequent treatment with TNF-α results in caspase-8-mediated cleavage of GSDMC, leading to pyroptosis and tumor necrosis in breast cancer.^36^ Furthermore, tumor cell ablation of mixed-lineage leukemia 4 (MLL4) was found to activate the transcriptional reactivation of GSDMD-dependent pyroptosis through enhancer decommissioning and oncolytic parapoxvirus ovis (ORFV) and was shown to pre-stabilize GSDME by mitigating its ubiquitination and subsequently inducing tumor cell pyroptosis.^330,380^ Additionally, USP48 stabilizes GSDME by removing K48-linked ubiquitination at K120 and K189, thereby promoting pyroptotic death in cancer cells.^150^

**Fig. 5:**
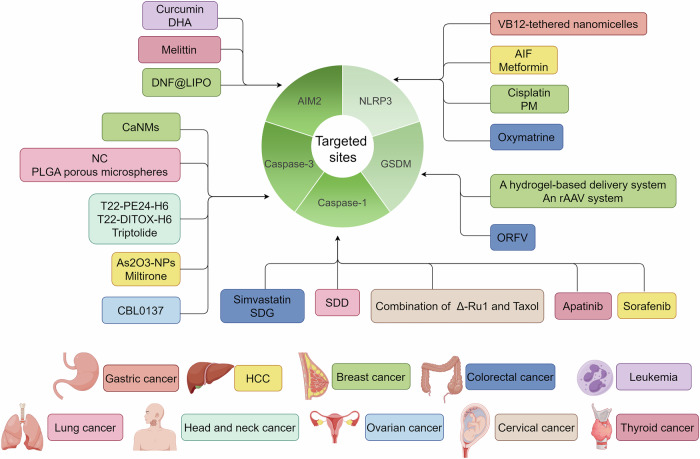
Potential cancer treatment strategies that target pyroptosis. Tumor cells undergoing pyroptosis release immunogenic substances and pro-inflammatory cytokines that promote immune cell activation and recruitment. This may engage in a positive feedback loop that augments tumor-specific immunity and further increases tumor-specific immunity. Thus, recent research has examined the therapeutic viability and potential of manipulating pyroptosis as an anticancer treatment. Key components in the process, such as NLRP3, AIM2, caspase-1, caspase-3, and gasdermins, have been developed into several specific therapeutic agents for malignancies. The figure was created by Figdraw

These innovative and diverse approaches demonstrate the potential of targeting GSDM and the pyroptotic pathway in anti-tumor therapeutic strategies, offering promising avenues for further exploration and development, as summarized in Fig. 5 and Table 2.

**Table 2:** Summary of agents targeting molecules of pyroptosis in anti-cancer therapeutics

Classification	Drug	Mechanisms	Cancer type	References
Small compound	FL118	NLRP3/Caspase-1/GSDMD	Colorectal cancer	^336^
NPs	VB12-sericin-PBLG-IR780	ROS/NLRP3/Caspase-1/GSDMD	Gastric cancer	^347^
Small compound	AIF	NLRP3/Caspase-1/GSDMD	HCC	^334^
antidiabetes drug	Metformin	FOXO3/NLRP3/Caspase-1/GSDMD	HCC	^335^
FDA-approved drug	Cisplatin	MEG3/NLRP3/Caspase-1/GSDMD	TNBC	^338^
Small compound	A438079	NLRP3/Caspase-1/GSDMD pathway by inhibiting P2X7R	Colorectal cancer	^153^
Small compound	Curcumin	ISG3/AIM2/Caspase-1/GSDMD, ISG3/IFI16/Caspase-1/GSDMD, ISG3/NLRC4/Caspase-1/GSDMD	Acute myeloid leukemia	^348^
NPs	DNF@LIPO	AIM2/Caspase-1/GSDMD	Breast cancer	^351^
Antimalarial drug	DHA	AIM2/Caspase-3/GSDME	Breast cancer	^349^
NPs	PLGA porous microspheres	Caspase-3/GSDME	Lung cancer	^363^
NPs	T22-PE24-H6 and T22-DITOX-H6	Caspase-3/GSDME	HNSCC	^364^
NPs	CaNMs	ROS/Caspase-3/GSDME	Breast cancer	^352^
NPs	As_2_O_3_-NPs	Caspase-3/GSDME	HCC	^365^
Small compound	Triptolide	Caspase-3/GSDME	Head and neck cancer	^355^
Small compound	Gambogic acid	Caspase-3/GSDME	Colorectal cancer	^356^
Small compound	Miltirone	ROS/Bax/Caspase-3/GSDME	HCC	^357^
Small compound	Tetraarsenic hexoxide	ROS/Caspase-3/GSDME	TNBC	^358^
FDA-approved drug	Cisplatin and paclitaxel	Caspase-3/GSDME	Lung cancer	^105^
Chinese medicine	Cordyceps militaris extract	Caspase-3/GSDME	Lung cancer	^362^
Small compound	Nitidine chloride	Caspase-3/GSDME pathway by suppressing PI3K/Akt pathway	Lung cancer	^359^
FDA-approved drug	Sorafenib	Caspase-1/GSDMD	HCC	^374^
NPs	A hydrogel-based delivery system carrying calcium chelator	GSDMD	Breast cancer, melanoma, and ovarian cancer	^378^
NPs	Sf9/rBac system and Cre/lox system	GSDMD	Glioblastoma, breast cancer and TNBC	^379^
Oncolytic virus	ORFV	GSDME	Melanoma	^380^

### Neurological disease

#### Alzheimer’s disease

AD is distinguished by its neuropathological features, such as amyloid-β plaques outside cells, neurofibrillary tangles inside cells, neuroinflammation, and loss of neurons.^165^ Targeted suppression of inflammasome-dependent pyroptosis alleviates AD-related symptoms. Sodium houttuyfonate (SH) suppressed the expression of NLRP3 and cleavage of GSDMD, ameliorated pyroptosis in hippocampal neurons, and mitigated deficits in spatial learning and memory in mice with AD induced by Aβ1-42.^381^ L7 (derivative of N-salicyloyl tryptamine) counteracts pyroptosis in BV2 cells triggered by Aβ through the inhibition of the NLRP3-caspase-1 signaling cascade, thereby offering neuroprotection through reduced GSDMD expression.^382^ 1,7-diphenyl-4-hepten-3-one (C1), a natural diarylheptanoid, was found to alleviate AD-like pathology by suppressing pyroptosis via activation of the Nrf2 pathway, thus downregulating the expression of NLRP3, GSDMD, and caspase-1.^383^ Telmisartan, a widely used anti-hypertensive drug approved by the FDA, can further ameliorate inflammatory effects via the inhibition of the microglial PPARγ/NLRP3 pathway.^384^ Additionally, artificial silencing of the *INPP5D* gene could promote NLRP3 production in microglia, providing therapeutic potential for AD treatment.^385^ The selective blockade of NLRP1, caspase-1, and caspase-6 ameliorates neuroinflammation and cognitive deficits in transgenic mice with AD.^386^ Chinese traditional medicine acupuncture also exhibits similar treatment efficiency. The Bushen Huoxue acupuncture technique diminishes Aβ generation in the hippocampal tissue of SAMP8 mice, suppresses NLRP1 inflammasome activation-medicated pyroptosis, and ultimately enhances cognitive function in mice with AD.^387^ Specific blockades of caspase-GSDM-mediated pyroptosis have demonstrated noteworthy neuroprotective effects in animal models of AD. In experiments involving APP/PS1 mice, mafenide (MAF) derivatives restrained GSDMD activation-induced pyroptosis and neuroinflammation by impeding cleavage at the GSDMD-Asp275 site.^388^ Another study showed that inhibition of inflammasome activation by MCC950, an NLRP3 inhibitor, improved cognitive function in APP/PS1 mice.^170^

Preserving BBB integrity through suppression of pyroptosis diminishes Aβ aggregation. Research has indicated that inflammatory mediators released from pyroptotic neurons during cerebrovascular disease significantly undermine BBB integrity.^389^ Traumatic brain injury (TBI) triggers pyroptosis in impaired brain microvascular endothelial cells (BMVECs) via NLRP3 inflammasome activation. The caspase-1 inhibitor AcYVAD-CMK can impede NLRP3 inflammasome activation by preventing GSDMD cleavage and ASC oligomerization, thus preserving the integrity of the BBB.^330^

#### Parkinson’s disease

PD symptoms, similar to those of AD, can be alleviated by targeting the suppression of inflammasome-dependent pyroptosis. A small molecular NLRP3 inhibitor, MCC950, inhibited α-synuclein-mediated inflammasome activation, attenuating nigrostriatal dopaminergic degeneration and motor deficits.^176^ Salidroside (Sal) safeguards dopaminergic neurons by attenuating NLRP3-dependent pyroptosis via (1) indirectly diminishing the synthesis of NLRP3, pro-IL-1β, and pro-IL-18 through the inhibition of the TLR4/MyD88/NF-κB signaling cascade, and (2) directly inhibiting pyroptosis by targeting the TXNIP/NLRP3/caspase-1 signaling pathway.^390^ Additionally, synchronized upregulation of pyroptosis protein expression and Parkinson’s-like symptoms in 6-hydroxydopamine-induced PD rat models can be inhibited by kaemperfol via the p38MAPK/NF-κB signaling pathway.^391^ Experimental data from both in vitro and in vivo studies have revealed that the Prussian blue nanozyme (PBzyme) mitigates the activation of microglial NLRP3 inflammasomes and caspase-1 by neutralizing ROS. This action results in reduced cleavage of GSDMD and decreased production of inflammatory mediators, culminating in the suppression of microglial pyroptosis.^392^ In both in vivo and in vitro PD models, β-hydroxybutyrate (BHB) supplementation suppresses pyroptosis by attenuating the activation of the NLRP3 inflammasome via downregulation of STAT3-mediated signaling.^393^ Traditional Chinese drugs also exhibited the ability to regulate pyroptosis. Qiji Shujiang granule (QJG) mediates the reduction of pyroptosis by inhibiting the NLRP3/caspase-1 axis, thereby conferring a neuroprotective benefit.^394^

Targeting the upstream molecules of the pyroptosis pathway is another option. TLR4 has been shown to sense LPS and induce pyroptosis through a signaling cascade.^395,396^ TLR4 cell-surface expression, LPS sensing, dimerization, and signaling depend on TLR4 binding to MD-2.^397^ Disulfiram (DSF) was found to modify Cys133 of MD-2 and inhibit TLR4 sensing, suppressing neuroinflammation and dopaminergic neuron loss.^398^

#### Stroke

Stroke remains the predominant cause of mortality and persistent impairment globally, with ischemic stroke accounting for approximately 85% of total occurrences.^399^ Targeting the NLRP3 inflammasome to inhibit pyroptosis has been the dominant strategy for alleviating stroke symptoms in the past few years. Pinocembrin, a natural product, was discovered to inhibit the activation of the TLR4/NF-κB pathway, suppressing the downstream assembly of the NLRP3 inflammasome and ameliorating vascular lesions.^400^ RRx-001 shows promise as a specific inhibitor of NLRP3, primarily exerting its effects by binding to cysteine 409 of NLRP3 to disrupt the NLRP3-NEK7 interaction, which is crucial for NLRP3 activation. RRx-001, currently undergoing phase III clinical trials, has a reasonably good safety profile and is a promising therapeutic candidate for stroke.^401^ Meanwhile, inhibition of Janus kinase can ameliorate ischemic stroke injury and neuroinflammation through reducing NLRP3 inflammasome activation via JAK2/STAT3 pathway inhibition.^402^ More drugs, such as lonidamine, edaravone dexborneol, thiolutin, and β-1, 3-galactosyltransferase-2, may inhibit NLRP3 assembly. However, few of these drug candidates could be translated into clinical research.^403–406^

#### Multiple sclerosis

The NLRP3 inhibitors JC-171, OLT1177, and MCC950 can efficiently ameliorate EAE pathogenesis, improve deficient symptoms, and prevent cognitive deficits in patients with MS.^407–409^ Pyroptosis was inhibited by the caspase-1 inhibitor, VX-765, in an EAE model. VX-765 treatment improved neurobehavioral performance and reduced pyroptosis-related protein expression and axonal injury.^180,409^

### Respiratory diseases

#### Asthma

Yanghe Pingchuan has been used to treat asthma for many years in China. While the detailed mechanism remains unknown, Yanghe Pingchuan was discovered to inhibit airway smooth muscle cell pyroptosis in asthma by suppressing the TLR4/NF-κB/NLRP3 signaling pathway.^410^ JT002 treats asthma by inhibiting NLRP3 assembly, thus alleviating airway hyperresponsiveness and neutrophilia.^411^ Likewise, heme oxygenase-1 (HO-1), an enzyme inducible for degrading heme, was shown to inhibit GSDMD-mediated pyroptosis and release of cytokine TSLP in airway epithelial cells by interacting with the RHD domain of NF-κB p65 and modulating NF-κB-dependent pyroptosis.^412^ Furthermore, additional NF-κB/NRLP3-mediated asthma treatment candidates include Schisandrin B and Protopine.^413,414^

#### Chronic obstructive pulmonary disease

COPD, a prevalent condition that can be prevented and managed, is characterized by persistent respiratory symptoms and restricted airflow resulting from airway and alveolar anomalies. Several pyroptosis inhibitors have been explored to treat COPD, with most targeting the NLRP3 inflammasome. Schisandrin A, a lignan of the diphenyl cyclooctadiene class extracted from *Schisandra chinensis* fruits, possesses a range of pharmacological activities; it mitigates ROS generation and NLRP3 inflammasome activation through the upregulation of Nrf-2, thus exerting anti-inflammatory effects and diminishing pulmonary damage in mouse models of COPD.^415^ Tian et al. discovered that (-)-epicatechin, a flavonoid compound, suppresses NLRP3 inflammasome activation by enhancing Nrf2 activity and alleviating lung inflammation triggered by cigarette smoke, which was validated through the reduced secretion of IL-1β and IL-18 in a rat model of COPD.^416^ Similarly, propofol, an anesthetic agent, increases Nrf2 expression and inhibits NLRP3 expression.^417^

In addition, physical or support methods can alleviate pyroptosis in COPD. For instance, halotherapy was found to alleviate oxidative stress in the lung tissues of COPD rats, diminish the accumulation of CD4^+^ and CD8^+^ T cells in the lungs, and reduce the production of inflammatory factors in the serum by suppressing the TLR4/NF-κB/GSDMD and NLRP3/ASC/caspase-1 pathways.^418^ The success of halotherapy in alleviating pyroptosis in patients with COPD provides incentives for the more detailed exploration of physical treatments in respiratory diseases.

#### Acute lung injury

Several molecules have been found to improve ALI symptoms by regulating pyroptosis, most of which target the upstream molecules of the NLRP3 inflammasome. For example, buformin, a hypoglycemic agent, can drive phosphorylation of AMPK, inhibit downstream NLRP3 inflammasome production, and accelerate autophagy, which, in turn, promotes NLRP3 inflammasome degradation, inducing a therapeutic effect after ALI.^190^ Emodin, an anthraquinone compound derived from rhubarb, and *Polygonum cuspidatum* treat AP-associated ALI by regulating macrophages and neutrophils via the targeting of NLRP3 production. Emodin mitigates the pyroptotic process of alveolar macrophages by decreasing the level of inflammatory cytokines and lactate dehydrogenase via NLRP3 inhibition and cold-inducible RNA-binding protein-activated NLRP3/IL-1β/CXCL1 signaling to dampen neutrophil infiltration.^419,420^ Furthermore, dehydroandrographolide, sourced from the traditional Chinese herb *Andrographis paniculata*, mitigates NLRP3-mediated pyroptosis in acute lung injury models by causing ROS-induced mitochondrial damage, which is achieved through the suppression of the Akt/Nrf2 signaling pathway, mediated by PDPK1 ubiquitination.^421^ Similarly, chicoric acid effectively inhibited pyroptosis by inducing mitochondrial damage via ROS generation. This effect is mediated by the activation of the Akt/Nrf2 pathway via PDPK1 ubiquitination.^422^ Other potential drugs regulate pyroptosis by targeting distinct pathways. Tangeretin mitigates ALI induced by sepsis by suppressing ROS-mediated activation of the NLRP3 inflammasome by modulating the PLK1/AMPK/DRP1 signaling axis.^423^ EuHD1 hinders the formation and activation of the NLRP3 inflammasome by suppressing ROS production and ASC oligomerization.^424^ Ren et al. revealed that the anti-pyroptotic effect of ergolide is conferred through direct targeting of the NACHT domain of NLRP3.^425^ Finally, britannin is an effective and natural NLRP3 inhibitor.^426^ The diversity of upstream molecules of NLRP3 offers various choices, but the interactions between these molecules and which one possesses the dominant role remain to be determined.

Other targets of pyroptosis have the potential to treat ALI. Zhang et al. found that Xuebijing, a traditional Chinese medicine recognized for its potent anti-inflammatory properties, inhibited gasdermin-E-mediated pyroptosis of lung cells by suppressing TNF-α production.^427^ Moreover, targeting pyroptosis has therapeutic implications for other respiratory diseases. For example, targeting caspase-1 by tetracycline blocks pyroptotic cell death in macrophages exposed to silica particles during silicosis.^428^ Colchicine inhibits NLRP3 inflammasome and inflammation to ameliorate COVID-19 pneumonia which are verified in clinical trials.^429^

In summary, targeting the NLRP3 inflammasome is a viable strategy for inhibiting pyroptosis and treating respiratory diseases, whereas other targets, such as caspase-1, also exhibit therapeutic potential.

### Kidney diseases

#### Acute kidney injury

Similarly, the NLRP3 inflammasome serves as a potential target for AKI treatment. The carboxy-terminus of Hsc70-interacting protein (CHIP), a U-box E3 ligase, is known to modulate oxidative stress by degrading its target proteins and has been found to engage with NLRP3, ubiquitinating it to facilitate its degradation via the proteasome, thereby suppressing pyroptosis mediated by the NLRP3/ASC inflammasome.^430^ The Klotho protein, which is beneficial in AKI, including for anti-senescence, anti-oxidation, anti-inflammation, and anti-fibrosis, inhibited the NLRP3 inflammasome by promoting cell autophagy, improving AKI.^431^ miR-30c-5p can suppress the expression of NLRP3, ASC, and caspase-1 by directly interacting and inhibiting TXNIP.^432^ Additionally, circ DENND4C, secreted by urine-derived stem cells in the form of exosomes, interacts with the miR 138-5p/FOXO3a axis, inhibiting the NLRP3 inflammasome.^433^ In addition to the NLRP3 inflammasome, caspase-1 is another candidate target for pyroptosis inhibition. Carnosine, a dipeptide known for its antioxidant and anti-inflammatory effects, reduces damage in kidney tubular epithelial cells by targeting caspase-1 and suppressing caspase-1/GSDMD-driven pyroptosis.^434^ Although these targets are embedded upstream of the pyroptosis pathway, GSDMD, an executor of pyroptosis, exhibits therapeutic potential. Recent studies have highlighted that dual-specificity phosphatase 2 (DUSP2) is a pivotal modulator of cell death and inflammation in several diseases. Functioning as a nuclear phosphatase, DUSP2 deactivates STAT1, a transcriptional suppressor of GSDMD, thereby limiting GSDMD-mediated pyroptosis in renal tubular epithelial cells.^435^

#### Diabetic kidney disease

Three PubMed-indexed studies have targeted pyroptosis for DKD treatment, and all targeted NLRP3-mediated inflammation. Loganin mitigates pyroptosis in HK-2 cells triggered by high glucose levels by suppressing ROS generation and NLRP3 inflammasome activation, resolving renal pathologies in DKD mice, similar to ManNAc in podocytes.^436,437^ Likewise, the anti-pyroptotic effect of the Tangshen formula acts via the TXNIP-NLRP3-GSDMD axis.^438^

#### Lupus nephritis

Honokiol, derived from the bark of *Magnolia officinalis*, is a versatile lignan with diverse pharmacological effects, including anti-inflammatory, antioxidant, and anti-tumor properties, with minimal side effects.^439^ Ma et al. unveiled that honokiol can suppress the renal activation of the NLRP3 inflammasome in macrophages, inhibiting the release of IL-33 and IL-1β and avoiding renal tubular epithelial cell death during lupus nephritis.^440^ Additionally, quercetin, a bioactive compound naturally occurring in various plants, has been identified for its role in improving pyroptosis mediated by inflammasomes and GSDMD. This effect is primarily attributed to the regulation of the IL-33/ST2 pathway and potentially involves the IL-33/TLR4 pathway in renal tubular epithelial cells.^441^

### Cardiovascular diseases

#### Atherosclerosis

Pyroptosis has been a treatment target for atherosclerosis, as exemplified by the candidate molecule salvianolic acid A in diabetic atherosclerosis.^442^ Typically, apigenin, lncRNA H19, organogermanium compound 3-(trihydroxygermyl) propanoic acid (THGP), and Z-LLSD-FMK or Z-YVAD-FMK improve atherosclerosis by targeting NF-κB, caspase-1, caspase-1, and GSDMD, respectively.^443–446^ However, some molecules regulate pyroptosis through distinct targets. Melatonin, a neuroendocrine hormone produced in the pineal gland and other organs, has been shown to suppress macrophage pyroptosis in atherosclerosis by downregulating the SIRT3/FOXO3α/ROS pathway, consequently diminishing caspase-1 dependent pyroptosis.^262^ Estrogen was reported to recognize estrogen receptor α and promote autophagy of endothelial cells, thereby downregulating the expression of NLRP3, cleaved caspase 1, and GSDMD.^265^ Likewise, Liu et al. revealed that blocking the p62/Nrf2/ARE signaling pathway using chloroquine through autophagy impairment can promote pyroptosis in macrophages, providing a novel therapeutic target for atherosclerosis treatment.^447^ Thus, the MALAT1/miR-30c-5p/Cx43 axis is a potential target for atherosclerosis therapy. Yang et al. revealed that the expression of MALAT1 and Cx43 was upregulated, while miR-30c-5p was downregulated in rat aortic endothelial cells during pyroptosis following atherosclerosis. Furthermore, MALAT1/miR-30c-5p/Cx43 comprises a signal cascade that regulates pyroptosis.^448^

#### Myocardial infarction

In recent years, numerous drugs and molecules have been explored to treat myocardial injury. Downstream regulation of NLRP3 expression has been the prominent target, by focusing on various upstream molecules. For example, geniposide promotes AMPK phosphorylation, Tanshinone IIA and Qighen granule suppress the TLR4/NF-κB p65 signaling pathway, hydrogen gas inhalation reduces the production of ROS, and chlorogenic acid depresses lncRNA Neat1 expression, ultimately impacting cardiomyocyte pyroptosis.^449–453^ In clinical trials, coenzyme Q10 is verified to suppress the recruitment of pro-inflammatory CCR2^+^ macrophages by attenuating the activation of the NLRP3/ IL-1β pathway, ameliorating MI.^454^ The AIM2 inflammasome also exhibits therapeutic potential in MI. Epigallocatechin-3-gallate, a bioactive polyphenol isolated from green tea, protects cardiomyocytes from pyroptosis via the MEG3/TAF15/AIM2 axis, thereby inhibiting AIM2 expression.^455^ Moreover, other targets in the canonical pathway, such as GSDMD, have gained attention in MI. Danhong, a traditional Chinese medicine, injection directly blocks GSDMD-N oligomerization and pore formation.^101^ MicroRNA-182-5p, carried by MSC-derived exosomes, also directly targets GSDMD and suppresses pyroptosis.^456^ In addition to the direct effects of upstream molecules on the NLRP3 inflammasome, additional targets have been of interest. Magnetic stimulation therapy also contributes to the treatment of MI. Lu et al. found that magnetic vagus nerve stimulation inhibited cardiomyocyte pyroptosis by activating M_2_AChR to suppress oxoglutarate dehydrogenase-like expression.^457^ In summary, various targets are involved in the regulation of pyroptosis in MI treatment.

#### Diabetic cardiomyopathy

Pyroptosis-inhibitory compounds targeting upstream molecules of NLRP3 have been discovered in the context of DCM. For example, the bone morphogenetic protein-7 cascade represses the expression of Nek7, GBP5, and TLR4, eventually inhibiting NLRP3.^458^ Metformin can activate the AMPK/mTOR pathway, thereby improving cell autophagy and disrupting NLRP3-mediated pyroptosis.^459^ Similarly, berberine suppresses NLRP3 by disturbing mTOR/mtROS.^460^ Fufang Zhenzhu Tiaozhi, a Chinese herbal medicine, and pomegranate peel extract exhibit cardioprotective potential through NLRP3 inhibition.^461,462^ GSDMD is also a target for DCM treatment. Lu et al. discovered that the protective role of irisin and mitochondrial ubiquitin ligase (MITOL/MARCH 5) in DCM was partially offset by the activation of cGAS/STING signaling, inhibiting GSDMD-mediated pyroptosis.^463^ Targeting Nrf2 has a pivotal role in inhibiting pyroptosis. For instance, curcumin can reduce the accumulation of superoxide in the myocardium through AKT/Nrf2/ARE pathway activation and inhibit pyroptosis, promoting nuclear translocation of Nrf2, increasing expression of antioxidant factors in cells and inhibiting the progression of cell pyroptosis.^464,465^ Similarly, puerarin and quercetin can inhibit pyroptosis by regulating P2X7 receptor expression. However, these two compounds exhibit opposite effects on the expression of the P2X7 receptor, leaving the mechanism of pyroptosis inhibition to be elucidated.^465,466^

### Digestive diseases

#### Inflammatory bowel disease

Xu et al. proposed a novel approach using self-adaptive pyroptosis-responsive liposomes to treat autoimmune inflammatory diseases, including inflammatory bowel disease. Following pyroptosis, the activated GSDME-N bound to the cardiolipin on the liposome surface, forming pores releasing encapsulated dimethyl fumarate and inhibiting the caspase 3/GSDME pathway.^467^ A PLGA-microsphere-carried circGMCL1 was designed to protects against Crohn’s colitis through suppressing NLRP3 inflammasome-dependent pyroptosis via regulation of miR-124-3p/ANXA7-induced autophagy.^468^ Human umbilical cord mesenchymal stem cell-derived exosomes were found to protect against colitis via the regulation of macrophage pyroptosis in a miR-378a-5p-denpendent manner.^469^ Furthermore, β-sitosterol, Tou Nong powder, necrosulfonamide, Munronoid I, and ginsenoside Rg3 have been associated with inflammatory bowel disease treatment.^470–474^

#### Acute pancreatitis

The natural compound wedelolactone has been reported to impede AP progression and associated lung injury through the canonical caspase-1-mediated pyroptotic pathway and caspase-11-mediated non-canonical pyroptotic pathway.^475^ Li et al. demonstrated that hair follicle-derived mesenchymal stem cell-derived small extracellular vesicles alleviated AP by suppressing inflammation and pyroptosis in pancreatic acinar cells. Furthermore, compared to the effect of small extracellular vesicles administered intraperitoneally, the therapeutic effect of small extracellular vesicles following intravenous injection seems to be enhanced.^476^ Qingjie Huagong decoction, a formula consisting of seven traditional medicines, has been reported to exert its anti-AP effects by regulating the circHipk3/miR-193a-5p/ NLRP3 pathway.^477^ Other therapeutic strategies have also emerged to target pyroptosis in the pathology of AP, including baicalein, disulfiram, sinapic acid, and salidrosidet.^478–481^

#### Liver fibrosis

**Fig. 6:**
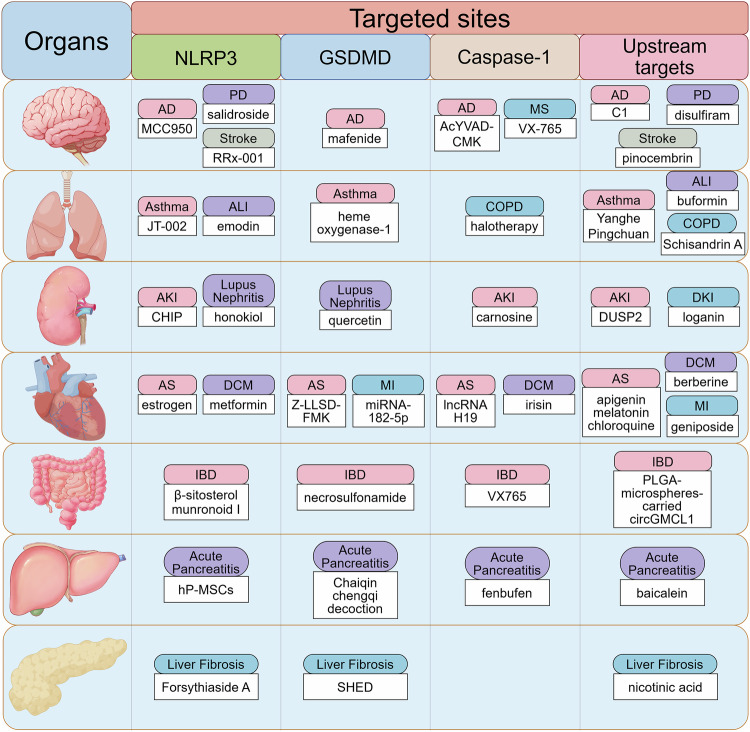
Potential targeted therapeutic strategies for pyroptosis in non-cancer diseases. Investigations into pyroptosis enhance our understanding of the pathological mechanisms underlying various diseases. Key elements involved in pyroptosis, including NLRP3, caspase-1, gasdermin D, and upstream molecules such as TLR4/NF-κB axis, are crucial for human health and have led to the development of numerous targeted therapies for several inflammatory conditions. The figure was created by Figdraw

Forsythiaside A (FA), a natural bioactive ingredient derived from traditional Chinese medicine, was loaded into a CD44-targeting exosome nanocarrier and mitigated liver fibrosis. The anti-liver fibrosis mechanism could be attributed to the suppression of NLRP3-mediated pyroptosis.^482^ Stem cells from human exfoliated deciduous teeth attenuated liver cirrhosis by suppressing the GSDMD-mediated pyroptosis pathway via decreasing ROS in hepatocytes.^483^ Nicotinic acid (NA), a vitamin used to treat dyslipidemia, was found to inhibit the NF/κB/NLRP3 signaling axis, thereby preventing pyroptosis and liver fibrosis during non-alcoholic steatohepatitis progression.^484^ Furthermore, researchers have identified various pioneering compounds that target pyroptosis for treating liver fibrosis, such as JT001, trilobatin, ursolic acid, and auranofin.^485–488^ Additionally, the clinical efficacy of some drugs was tested. A phase I clinical trial verified that emricasan inhibits excessive caspase activation and lowers ALT in patients with non‐alcoholic fatty liver disease, which may ameliorate the progression from fatty liver to liver fibrosis.^489^ These molecules and targets’ potential for non-cancer disease treatment are summarized and visualized in Fig. 6.

## Conclusion and perspective

Pyroptosis is a distinct form of cell death characterized by inflammasome formation, caspase-mediated gasdermin cleavage, and inflammatory cytokine release. As an RCD form, the ubiquity of pyroptosis in cells indicates its great promise and remarkable drug market potential of pyroptosis-targeted drugs. For example, in cancer cells, pyroptosis can induce regional inflammation and convert “cold tumors” to “hot tumors,” triggering enhanced antitumor immunity and sensitizing checkpoint blockade immunotherapy.^490^ Pyroptosis-targeted therapy can assist in antitumor immunotherapy by inducing tumor cell pyroptosis, similar to chemotherapy but with fewer side effects. Pyroptosis-targeted drugs are promising therapeutic methods for tumors in addition to classical chemo-, radio, and immunotherapies. In addition, the intraepithelial mast cell-driven gasdermin C-mediated type 2 immunity has also been identified recently, which can be exploited as a potential drug target for allergic diseases.^491^ Besides, studies focusing on pyroptosis have made significant progress in recent decades at multiple levels, including elucidation of signaling mechanisms, disease regulation, and molecular structures. NLRP3/caspase-1/GSDMD and caspase-3/GSDME are two main druggable pathways for pyroptosis-related diseases. Previous research widely reported that the therapeutic effect of some classical drugs and traditional Chinese medicines is partly mediated through pyroptosis pathways. For instance, the IL-1R inhibitor, Anakinra, can relieve liver inflammation by targeting NLRP3 inflammasome.^492^ Atorvastatin inhibits the expression of NLRP3 to prohibit the progression of atherosclerosis. As for targeting caspase, numerous natural substances such as dendrobine can inhibit the expression or maturation of caspase-1 to achieve therapeutic outcome.^493^ Most chemotherapy drugs such as doxorubicin (Dox) and cisplatin induce cancer pyroptosis by activating caspase-3. Furthermore, disulfiram, a drug for alcoholism treatment, can modify the Cys191 amino acid of GSDMD to inhibit its oligomerization, which is promising for inflammatory disease therapy.^396^ Therefore, large amounts of approved drugs possess the potential to regulate pyroptosis for therapy. Although drug innovation targeting pyroptosis pathways is hard because of the interlaced signal pathway and complicated molecular structures such as NLRP3 inflammasome, increasing small molecule drugs have been reported recently. For example, while UK5099 can selectively inhibit NLRP3 inflammasome, necrosulfonamide, a chemical inhibitor, can selectively bind to GSDMD inhibiting its oligomerization to serve as a potential drug for inflammatory diseases.^494,495^ Molecular structures identified by cryo-electron microscopy or artificial intelligence such as Alphafold 3 provide increasing structures for scientists to identify novel drug targets by molecular docking, experiment verification, and further clinical trials.^496^ However, several challenges remain regarding the research and development of pyroptosis drugs with potential therapeutic strategies.

Disease heterogeneity presents a formidable challenge for pyroptosis drugs. For example, tumor heterogeneity exists not only between but also within different malignancies. Variations in the expression and function of pyroptosis-related molecules among different tumors may affect the responsiveness of tumors to pyroptosis-inducing therapies. Crosstalk between pyroptosis and other signaling pathways also complicates the cytoplasmic microenvironment. Given the heterogeneous intracellular and tumor microenvironments, pyroptosis-based therapies are challenging. Identifying a suitable patient population for pyroptosis therapy is crucial for future clinical trials. Inspired by targeted therapy in patients with cancer based on molecular classification, data-driven patient selection is promising for improving the therapeutic efficacy of pyroptosis-based therapies. Integrating omics and clinical information may also help predict the therapeutic outcomes of pyroptosis drugs in patients. For example, the expression level of GSDME in cancer cells may predict the therapeutic response of GSDME-targeted therapy. Molecular classification for selecting suitable patients to receive pyroptosis-targeted therapy is the guarantee of a favorable outcome.

Second, effective and selective drugs that can safely induce or inhibit pyroptosis remain far from satisfactory. Research on pyroptosis-based drugs remains at the laboratory stage, with limited candidates entering the preclinical and clinical trial phases. These clinical trials are summarized in Table 3 among which butyrate and inulin, zinc supplementation, and dapansutrile ameliorate type 2 diabetes, Bechet’s disease, and gout by regulating pyroptosis, respectively.^497–499^ However, the safety concerns associated with the induction or inhibition of pyroptosis are paramount. Extensive inhibition of pyroptosis inevitably increases the probability of pathogen invasion which may bring unforeseen risks for patients. Off-target effects also deserve wide attention. For example, previous research demonstrated that GSDME activation is reliant on caspase-3 and that the expression level of GSDME is closely linked to the type of cell death induced by chemotherapeutic agents. Cells with high GSDME expression underwent pyroptosis in response to chemotherapy, whereas cells with low or absent GSDME expression experienced apoptosis. However, researchers also noted that GSDME expression is typically low in most tumor cell lines because of methylation of the GSDME gene promoter, while GSDME is broadly overexpressed in normal cell lines.^23,103^ Therefore, chemotherapy might also trigger caspase-3-mediated pyroptosis in normal cells with high GSDME expression, potentially contributing to the toxicity and side effects observed during chemotherapy. For instance, Zheng et al. demonstrated that the activation of the Bnip3-caspase-3-GSDME pathway following Dox treatment initiated GSDME-mediated pyroptosis, contributing to Dox-induced cardiotoxicity in vivo.^296^ Dox treatment results in the hyperactivation of the NLRP3 inflammasome and pyroptotic cell death in cardiomyocytes, which is a key mechanism underlying dilated cardiomyopathy in Dox-treated heart tissues. The absence of either NLRP3 or caspase-1 protects mice from Dox-induced dilated cardiomyopathy.^290^ The specificity of pyroptosis-targeted agents has emerged as a critical issue, emphasizing the need to minimize unintended consequences in healthy cells. Selecting the appropriate drug target, integrating nano-delivery and protein-degradation technologies, and combining other existing therapy strategies are viable pathways to improving therapeutic efficiency while mitigating the risk of toxicity. The identity of molecules’ structure helps to design and deliver drugs. The structure and related mechanism of NLRP1, NLRP3, GSDMB, and GSDMD have been analyzed by cryo-electron microscopy.^29,500–502^ Specific cell-targeted adeno-associated virus systems also possess the potential to precisely regulate pyroptosis. Furthermore, as immune cells can distinguish cancer cells from normal cells, pyroptosis-mediated immune mobilization by engineered immune cells such as CAR-T can selectively alter the tumor microenvironment, which can greatly reduce side effects.

**Table 3:** Selected clinical trials related to pyroptosis and the corresponding target

Drug	Signal pathway	Mechanism	Disease	References
Colchicine	NLRP3	Inhibit NLRP3 inflammasome activation and ‘cytokine storm’	COVID-19	^429^
Zinc supplementation	NLRP3/caspase-1	Inhibit NLRP3 and caspase-1 expression	Behçet’s disease	^498^
Butyrate and Inulin	TLR2/NLRP3/caspase-1 NF-κB1/NLRP3/caspase-1	Upregulate miR-146a and miR-9 hence inhibiting TLR2 and NF-κB1	Type 2 diabetes	^497^
Coenzyme Q10	NLRP3/ IL-1β	Suppress the recruitment of macrophages by inhibiting NLRP3/ IL-1β pathway	Myocardial infarction	^454^
Apatinib and Melittin	caspase-1/GSDMD AIM2/caspase-3/GSDME	Apatinib and melittin improve caspase‐1–GSDMD and AIM2-caspase‐3–GSDME pyroptosis, respectively	Anaplastic thyroid cancer	^353^
CAR-T cell	Caspase-3/GSDME	Release perforin and GzmB to induce tumor cell caspase-3-GSDME pyroptosis	CD19^+^ relapsed or refractory B cell leukemia	^37^
Dapansutrile	NLRP3/IL-6, IL-1β	Inhibit NLRP3 inflammasome thus hindering the cleavage of IL-6 and IL-1β	Gout	^499^

Finally, robust preclinical and clinical trials are warranted to determine the clinical applications of pyroptosis-based drugs. In addition to classical cell lines and primary cells from patients, organoids are novel approaches to evaluate drug effectiveness and safety before entering clinical trials. Organoids derived from adult and pluripotent stem cells serve as crucial preclinical models for cancer research and therapy development.^503^ For instance, Zhou et al. found that NLRP3 inflammasome facilitates silica-induced injury to lung epithelial cells and abnormal regeneration in lung stem/progenitor cell-derived organotypic models.^504^ In addition to in vitro studies, multi-species in vivo animal model verification is the cornerstone of further clinical trials. For example, MCC950, an NLRP3 inhibitor, is discovered to function in neurodegenerative diseases and myocardial infarction (MI) in mice models.^505^ Meanwhile, NLRP3-inflammasome inhibition reduces infarct size and preserves cardiac function in a pig MI model.^506^ Finally, unintended consequences, long-term effects, and a balance between therapeutic benefits and potential risks are issues that need to be addressed in further clinical trials. Therefore, a complete verification chain of in vitro-in vivo-clinical trials is the basis for the clinical translation of pyroptosis-targeted drugs.

In conclusion, scientific progress has pushed us into an exciting era in the field of pyroptosis research. Addressing the previously mentioned challenges is imperative to realizing the full potential of pyroptosis in clinical practice. We expect that the successful translation of pyroptosis-targeted therapy into clinical treatment will soon be a reality.
